# Discovery of a Novel Class of Covalent Dual Inhibitors Targeting the Protein Kinases BMX and BTK

**DOI:** 10.3390/ijms21239269

**Published:** 2020-12-04

**Authors:** Michael Forster, Xiaojun Julia Liang, Martin Schröder, Stefan Gerstenecker, Apirat Chaikuad, Stefan Knapp, Stefan Laufer, Matthias Gehringer

**Affiliations:** 1 Department of Pharmaceutical/Medicinal Chemistry, Institute of Pharmaceutical Sciences, Faculty of Sciences, University of Tübingen, 72076 Tübingen, Germany; michael.forster@uni-tuebingen.de (M.F.); xiaojun-julia.liang@uni-tuebingen.de (X.J.L.); stefan.gerstenecker@uni-tuebingen.de (S.G.); stefan.laufer@uni-tuebingen.de (S.L.); 2 Cluster of Excellence iFIT (EXC 2180) ‘Image-Guided & Functionally Instructed Tumor Therapies’, University of Tübingen, 72076 Tübingen, Germany; 3 Structural Genomics Consortium, Goethe University Frankfurt, Buchmann Institute for Molecular Life Sciences, Max-von-Laue-Straße 15, 60438 Frankfurt am Main, Germany; m.schroeder@pharmchem.uni-frankfurt.de (M.S.); chaikuad@pharmchem.uni-frankfurt.de (A.C.); knapp@pharmchem.uni-frankfurt.de (S.K.); 4 Institute of Pharmaceutical Chemistry, Goethe University Frankfurt, Buchmann Institute for Molecular Life Sciences, Max-von-Laue-Straße 9, 60438 Frankfurt am Main, Germany; 5 Frankfurt Cancer Institute (FCI) and German Translational Cancer Network (DKTK) Site Frankfurt/Mainz, 60438 Frankfurt am Main, Germany; 6 Tübingen Center for Academic Drug Discovery (TüCAD2), 72076 Tübingen, Germany

**Keywords:** tyrosine kinases, bone marrow tyrosine kinase on chromosome X, Bruton’s tyrosine kinase, Janus kinase 3, covalent inhibitors, chemical probes

## Abstract

The nonreceptor tyrosine TEC kinases are key regulators of the immune system and play a crucial role in the pathogenesis of diverse hematological malignancies. In contrast to the substantial efforts in inhibitor development for Bruton’s tyrosine kinase (BTK), specific inhibitors of the other TEC kinases, including the bone marrow tyrosine kinase on chromosome X (BMX), remain sparse. Here we present a novel class of dual BMX/BTK inhibitors, which were designed from irreversible inhibitors of Janus kinase (JAK) 3 targeting a cysteine located within the solvent-exposed front region of the ATP binding pocket. Structure-guided design exploiting the differences in the gatekeeper residues enabled the achievement of high selectivity over JAK3 and certain other kinases harboring a sterically demanding residue at this position. The most active compounds inhibited BMX and BTK with apparent IC_50_ values in the single digit nanomolar range or below showing moderate selectivity within the TEC family and potent cellular target engagement. These compounds represent an important first step towards selective chemical probes for the protein kinase BMX.

## 1. Introduction

The TEC kinase family constitutes the second largest family of nonreceptor tyrosine kinases including the five members Bruton’s tyrosine kinase (BTK), interleukin (IL)-2-inducible T-cell kinase (ITK or TSK/EMT), tyrosine kinase expressed in hepatocellular carcinoma (TEC), bone marrow tyrosine kinase on chromosome X (BMX or ETK) and tyrosine-protein kinase (TXK or RLK) [1]. All TEC family members except TXK commonly harbor an *N*-terminal pleckstrin homology (PH) domain required for membrane association via binding to phosphatidylinositols. The PH domain is followed by a TEC homology (TH) domain which includes a BTK-homology motif and two proline-rich regions in BTK, ITK and TEC, one of which is absent in BMX. In contrast to the other TEC family members, TXK is devoid of the PH and TH domains and membrane targeting is promoted by a palmitoylated cysteine string motif (CC) instead. All Tec family kinases also contain two SRC homology domains, SH3 and SH2, preceding the highly conserved C-terminal kinase domain [2]. This makes TEC kinases closely related to the SRC kinase family, which represents the largest family of nonreceptor tyrosine kinases.

**Figure 1:**
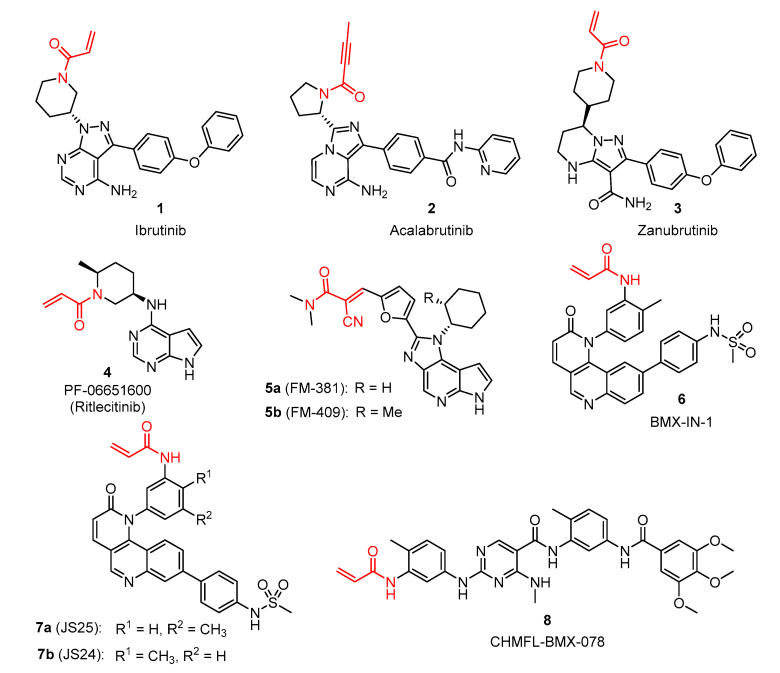
Examples of covalent Bruton’s tyrosine kinase (BTK), Janus kinase 3 (JAK3) and bone marrow tyrosine kinase on chromosome X (BMX) inhibitors including the FDA-approved BTK-targeted drugs ibrutinib, acalabrutinib and zanubrutinib. The electrophilic warhead group is highlighted in red.

TEC kinases differ in their expression pattern. While TEC and BMX are widely expressed in hematopoietic and endothelial cells, expression of BTK appears to be more restricted, mainly to myeloid and B-cells. On the contrary, ITK and TXK are predominantly expressed in T-lymphocytes [1]. Among the TEC kinases, BTK stands out as the best-studied family member due to its key function in B-cell receptor signaling required for B-cell development. Mutations in the *btk* gene are causative to human X-linked agammaglobulinemia (XLA) and murine X-linked immunodeficiency, which are characterized by a lack of mature B-lymphocytes and defective humoral immunity [3]. BTK is essential for the proliferation and survival of leukemic B-cells making this kinase not only a promising target for treatment of inflammatory and autoimmune diseases but also for B-cell malignancies. Currently, the three BTK inhibitors ibrutinib (**1**, Figure 1), acalabrutinib (**2**) and zanubrutinib (**3**) are approved for the treatment of various B-cell lymphoma and chronic graft-versus-host disease (only ibrutinib) and many more are in preclinical or clinical development [4,5,6]. Despite different selectivity profiles, these current drugs share a common mode of inhibition by covalently targeting BTK C481 located within the front pocket of the ATP binding site in the proximity of the αD helix. By comparison, a cysteine in this position exists also in other members of the TEC family and few additional kinases (*vide infra*).

Contrary to BTK, the effort on inhibitor development for other TEC kinases has remained rather limited despite several lines of evidence suggesting important roles in hematopoiesis as well as their potential as therapeutic targets. For instance, the function of ITK is crucial in T-cell receptor signaling. As such, it is required for T-cell development and response, controlling the production of proinflammatory cytokines [7]. ITK has primarily been considered as a target in the treatment of inflammatory and autoimmune diseases; however, so far, no selective ITK inhibitors have entered clinical development [4]. The role of other TEC kinases, including BTK’s closest relative BMX, in health and disease remains less clear. This highlights the need for specific chemical probes [8] to explore the biology of these understudied TEC kinases.

In recent years, targeting protein kinases with covalent inhibitors has become a mature strategy not only for the development of selective chemical probes, but also drugs [4,5]. Although protein kinases do not feature a catalytic nucleophile, a substantial fraction of human protein kinases are known to possess one or several accessible cysteine moieties inside or in close proximity to the ATP binding site [4,9,10,11]. These cysteines are located at diverse positions with a low degree of conservation. The mechanism of covalent binding typically follows a two-step process with an initial reversible binding event that positions the inhibitor’s “warhead” in the spatial proximity of a reactive amino acid residue [12]. High affinity noncovalent interaction along with suitable preorientation between the target kinase and the covalent inhibitor in the first step are essential for efficiency of the subsequent covalent bond formation, and hence on-target specificity [13]. Thus, covalent targeting of a poorly conserved amino acid residue may be employed as an additional selectivity filter complementing reversible recognition. Such a strategy has been very valuable in the context of protein kinase inhibitor design, where achieving selectivity is intrinsically challenging due to the high degree of conservation within this target class, especially in the ATP binding site [4,5]. Besides the possible advantages in terms of selectivity, covalent inhibitors possess other potential benefits. For example, persistent target occupancy enables an increased, time-dependent potency, lowering a hurdle on ATP competition. Such property may also decouple pharmacodynamics from pharmacokinetics, which can be particularly useful in a drug discovery setting [14]. Moreover, initial concerns about the safety of targeted covalent inhibitor drugs have not materialized so far [15]. Beyond drug discovery, the attachment of reporter tags (e.g., fluorescent dyes, affinity labels or click handles) to covalent ligands yields very useful tool compounds to explore various facets of target biology [13].

The presence of a nonconserved cysteine within the solvent-exposed front region at the N-terminus of the αD helix, often referred to as the αD-1 position [9], in all five TEC kinases (C496 in BMX; C481 in BTK) opens an opportunity for irreversible targeting. However, cysteines at the αD-1 position exist also in six other kinases, namely most members of the HER family (EGFR, HER2 and HER4), the Janus kinase JAK3, the SRC kinase BLK and the mitogen activated protein kinase kinase MKK7 (MAP2K7). Currently, the αD-1 cysteine has been targeted by seven approved drugs, including the EGFR/pan-HER inhibitors afatinib, dacomitinib and osimertinib, the HER2 inhibitor neratinib and the aforementioned BTK inhibitors ibrutinib (**1**), acalabrutinib (**2**) and zanubrutinib (**3**) [4,5]. Covalent BTK inhibitors often feature a certain cross-reactivity with other TEC family members. For example, ibrutinib shows strong off-target (re)activity in the entire TEC kinase family, but also for other kinases sharing the αD-1 cysteine [16] while acalabrutinib is more specific [17]. Covalent JAK3 inhibitors have attracted considerable interest [18,19] since the αD-1 cysteine (C909) is the key feature distinguishing JAK3 from the other JAK family members, JAK1, JAK2 and TYK2. For example, the acrylamide-based JAK3 inhibitor PF-06651600 (Ritlecitinib, **4**, Figure 1) [20] is currently being investigated in several phase II and III clinical trials. In our own JAK3 program, we generated highly selective covalent-reversible inhibitors FM-381 (**5a**) and FM-409 (**5b**) [21,22]. The equivalent cysteine in MKK7 (C218) is modified by hypothemicin analogs [4] but also by recently discovered acrylamide-based inhibitors [16,23,24].

As exemplified by BTK, irreversible inhibition represents an excellent strategy for targeting TEC kinases. Nevertheless, the development of inhibitors for other members of this family remains challenging due to the subtly different nature of their binding sites as well as the lack of understanding on these pockets. Design of specific ITK inhibitors, for example, is hindered by lower cysteine reactivity [25] and the larger gatekeeper residue. Only few reports describing the development of selective inhibitors of the less prominent TEC kinases, namely TXK, TEC and BMX, have been published so far. While no inhibitors addressing TXK or TEC as the primary target are known, two structural series of covalent BMX inhibitors targeting C496 have been disclosed. In 2013, Gray and colleagues identified the benzo[*h*] [1,6]naphthyridin-2(1*H*)-one derivative BMX-IN-1 (**6**, Figure 1), a dual covalent BMX/BTK inhibitor [26]. This compound has recently been complemented by a series of analogs from Bernardes and co-workers [27] (preprint on Chemrxiv), which feature increased potency but limited selectivity against JAK3, BLK and within the TEC family. JS25 (**7a**), the most potent BMX inhibitor from this set (IC_50_ = 3.5 nM, *k*_inact_/*K*_I_ = 19 µM^−1^s^−1^) showed increased cellular target engagement compared to **6** in a NanoBRET^TM^ assay [28] while covalent binding has been demonstrated for the close analog JS24 (**7b**) by mass spectrometry and crystal structure. A structurally distinct series of covalent BMX inhibitors has been published in 2017 by Liu and colleagues [29]. These compounds exemplified by CHMFL-BMX-078 (**8**) showed high affinity for the inactive (nonphosphorylated) kinase while affinity for the active (phosphorylated) kinase was weak (apparent *K*_d_ > 10 µM). It is assumed that the latter compound binds BMX and related kinases in a type II binding mode addressing the hydrophobic back-pocket sometimes referred to as “deep pocket” [30], which is only accessible in the DFG-out conformation. However, there is still a need for expanding the scope of novel BMX/TEC family kinase inhibitors with high selectivity or complementary selectivity patterns to study biology. Here we present a novel and structurally unrelated series of covalent BMX/TEC-family kinase inhibitors featuring a distinct selectivity profile among the kinases with a αD-1 cysteine.

## 2. Results

### 2.1. Discovery of a New Class of Covalent JAK3/TEC Family Kinase Inhibitors

**Figure 2:**
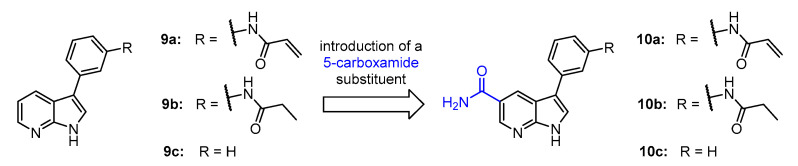
Design of covalent JAK3 inhibitor **10a** from analog **9a** by introduction of a carboxamide group at the 5-postion of the 7-azaindole core.

Our previous study demonstrated the successful use of the tricyclic imidazo [5,4-*d*]pyrrolo[2,3-*b*]pyridine scaffold for the design of selective covalent-reversible JAK3 probes FM-381 (**5a**, see Figure 1) and FM-409 (**5b**) targeting JAK3 C909 via an α-cyano acrylamide warhead [21,22]. Based on this, we aimed to expand the inhibitor series and explore their potentials as irreversible inhibitors for JAK3 and other kinases that harbor a cysteine at the equivalent position. With no success in generating acrylamide-derived irreversible inhibitors based on the aforementioned tricyclic scaffold, we tested alternative hinge binding motifs and synthesized 1*H*-pyrrolo[2,3-*b*]pyridine-derived compound **9a** (Figure 2, left)**.** This derivative retained excellent JAK inhibitory potency (IC_50_ = 0.2 nM) and high selectivity against the other JAK family members and EGFR, demonstrating the hinge binder replacement as a potential strategy for optimizing the parent inhibitor towards other kinases (Table 1).

**Figure 3:**
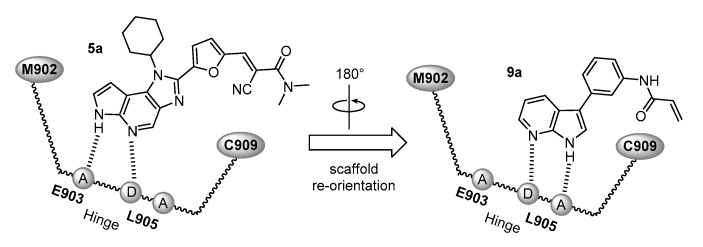
Differences in the hinge binding of different scaffolds exemplified by **5a** and **9a**. Usually, the protein backbone in the hinge region of a kinase provides three potential hydrogen bonding partners (two carbonyl-acceptors (A) and one NH-donor (D)). Depending on the orientation of the hinge binding heterocycle different hydrogen-bonding patterns are possible.

**Figure 4:**
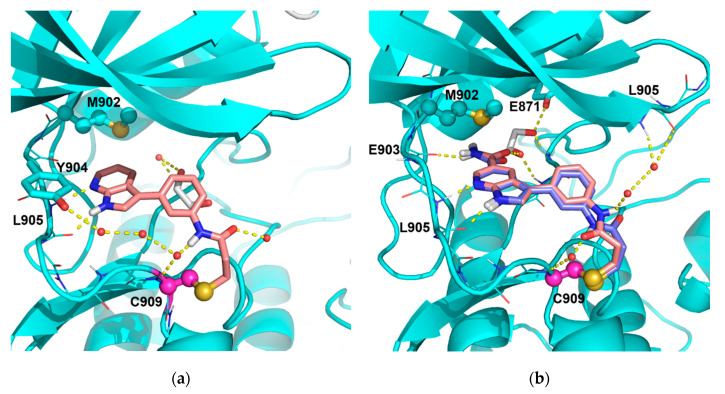
X-ray crystal structures of compounds **9a** (PDB-code 7APG) and **10a** (PDB-code 7APF) in complex with JAK3. (**a**) Compound **9a** covalently bound to JAK3-C909. An ethylene glycol molecule (depicted in gray) fills the space between the ligand and the DFG motif forming a hydrogen bond to D967 but no direct hydrogen bonds with the ligand. Certain water-mediated hydrogen bonds as well as an alternative pose with a flipped amide conformation were omitted for clarity; (**b**) Compound **10a** covalently bound to JAK3-C909. Two poses with a flipped warhead amide conformation were observed (depicted in salmon and blue). The carbonyl group of the 5-carboxamide moiety forms a hydrogen bond to an ethylene glycol molecule in the back pocket, which is further anchored by hydrogen bonding to E871 in the αC-helix and to the DFG motif. Certain water-mediated hydrogen bonds were omitted for clarity.

We hypothesized that the 1*H*-pyrrolo[2,3-*b*]pyridine (7-azaindole) core in **9a** might adopt an inverted binding orientation when compared to **5a**,**b** or common 7*H*-pyrrolo[2,3-*d*]pyrimidine derived JAK inhibitors like **4** with two hydrogen bonds between the 7-azaindole and the backbone of L905 (Figure 3). On this basis, we introduced a carboxamide group in the 5-position of the 7-azaindole scaffold to establish a third hydrogen bond towards the hinge region [32]. As expected, the resulting compound **10a** (Figure 2, right) exhibited an increased potency for JAK3 (IC_50_ < 0.1 nM) while maintaining a similar degree of selectivity against other JAKs. Interestingly, we observed that this compound demonstrated increased selectivity against EGFR compared to **9a**. In line with covalent binding, nonreactive analogs **9b**,**c** and **10b**,**c** showed a strongly decreased potency with JAK3 IC_50_’s in the micromolar range (not shown). The determined crystal structures of **9a** and **10a** (Figure 4) in complex with JAK3 confirmed the predicted hinge interaction pattern and covalent modification of C909. As expected, the 7-azaindole core in both compounds was anchored to the hinge region via two hydrogen bonds with the L905 backbone NH and carbonyl oxygen atom, respectively. Compound **10a** established an additional hydrogen bond between a carboxamide NH and the E903 backbone carbonyl oxygen atom. The second amide proton of the 5-carboxamide was involved in a presumably weak interaction with the methionine sulfur atom. The warhead amide was present in two flipped conformations (see Figure 4b) and involved in water-mediated hydrogen bonds, most notably with the backbone of C909. The latter interactions may assist in pre-orienting the Michael acceptor to facilitate cysteine addition.

To examine whether our replacement strategy of the hinge binding motif for expanding the profile of the JAK3 covalent inhibitors to other kinases succeeded, we characterized the activity of inhibitors **9a** and **10a** against all kinases featuring the αD-1 cysteine. This screening revealed that the compounds strongly inhibited BMX and TXK with no or less significant binding to other kinases at 200 nM (see Table 2). Notably, the observed selectivity pattern did not seem to rely on differences in the gatekeeper moiety which is methionine in JAK3 and MKK7 and a threonine in the HER and TEC family kinases (except ITK).

### 2.2. Evaluation of N-Substitution in the 5-Carboxamide Series

**Figure 5:**
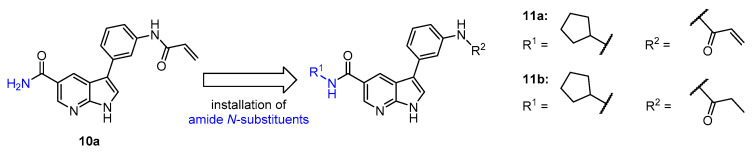
*N*-alkylation of the 5-carboxamide moiety.

To exploit the steric nature of the gatekeeper moiety as an additional selectivity filter, we introduced *N*-substituents at the 5-carboxamide’s nitrogen atom to preserve the triple hydrogen bonding interaction with the hinge region while forcing a steric clash with the bulky methionine gatekeeper in JAK3 or MKK7 and the phenylalanine in ITK. The first analog from this series with a relatively bulky *N*-cyclopentyl substituent (**11a**, Figure 5) did not show substantial activity at 200 nM on any of the kinases harboring an αD-1 cysteine. Nevertheless, we determined the IC_50_ values for JAK3, BMX and TXK to compare acrylamide **11a** with nonreactive propionamide **11b** (Table 3). While **11a** still showed residual activity on BMX, no JAK3 inhibitory activity was observed (IC_50_ > 10 µM). In contrast, analog **11b** did not inhibit any of the latter kinases up to 10 µM.

To test whether smaller *N*-substituents could prevent the loss in BMX potency, we prepared the corresponding *N*-methyl and *N*-ethyl analogs **11c** and **11d** (Table 4). In addition, we synthesized compounds **11e**–**g** to evaluate whether a methylene linker would be suitable to direct apolar cyclic moieties of variable size to the hydrophobic pocket behind the threonine gatekeeper often termed “hydrophobic region I” [34]. The binding of these derivatives was assessed using a thermal shift (∆T_m_) assay, which unfortunately revealed a dramatic decrease of ∆T_m_ for all compounds in relation to the starting point **10a** (Table 4). These results suggested that the applied modification strategy was not suitable to generate potent BMX inhibitors with high selectivity against JAK3.

### 2.3. Design and Structure**–**Activity Exploration of the 5-Acylamino Series

**Figure 6:**
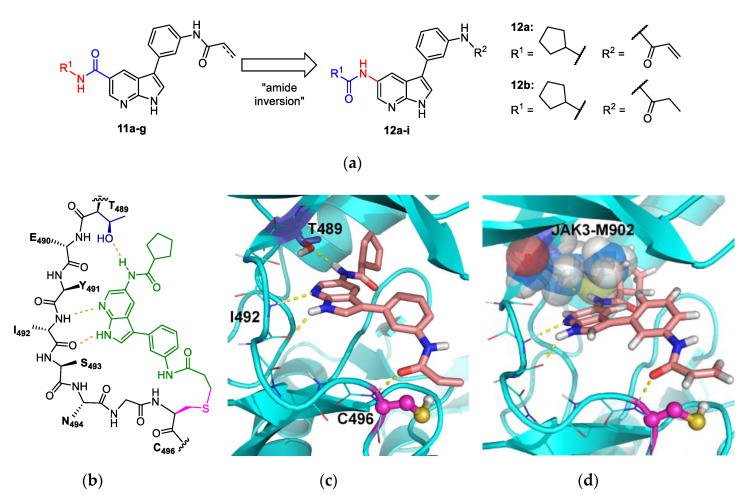
Amide inversion as a strategy to rescue BMX inhibitory activity. (**a**) General strategy of amide inversion and representative analogs **12a** and **12b**; (**b**) Schematic depiction of the expected binding mode of analog **12a**; (**c**) Representative noncovalent docking pose of compound **12a** modelled into the BMX active site using the BMX structure with the PDB-code 3SXR (BMX in complex with dasatinib) as a template. Similar poses were obtained using an alternative crystal structure (PDB-code 3SXS) in which the P-loop region is completely resolved. (**d**) Overlay of the docking pose of **12a** with the crystal structure of JAK3 (7APF) highlighting the clash with the JAK3 gatekeeper moiety. Only the gatekeeper methionine (transparent spheres) of the JAK3 structure is shown for clarity.

In search for alternative approaches exploiting the gatekeeper residue as a selectivity filter, molecular modeling studies were performed. Docking simulations suggested that inverting the 5-carboxamide substituent would enable a favorable NH···O hydrogen bond to the hydroxy group of the threonine gatekeeper in BMX. While the third hydrogen bond to the hinge region would not be compatible with this arrangement, the interaction with the gatekeeper was predicted to direct moieties attached at the carbonyl group towards the hydrophobic region I behind the gatekeeper moiety (see Figure 6).

We thus prepared a set of analogs with an *N*-linked amide moiety (compounds **12a**–**i**, see Figure 6 and Table 5) to validate this hypothesis. Satisfactorily, the compounds from this series showed increased BMX thermal shifts while JAK3 thermal shifts remained in a moderate range suggesting low inhibitory potency on the latter. To confirm these results, the IC_50_ values of a subset of compounds were determined for BMX and JAK3. In agreement, all tested inhibitors exhibited high inhibitory activities for BMX with IC_50_ values in a low nanomolar range. Moreover, all compounds displayed excellent selectivity against JAK3, which was most pronounced for the most active analog **12c** (>9000-fold selectivity).

We selected potent cyclopentanoic amide **12a** and compound **12c** with a thiophene 2-carboxylic acid amide moiety for further profiling against the kinases that have an equivalent cysteine at the αD-1 position. At a concentration of 200 nM, **12a** strongly inhibited BMX, BTK and TXK while TEC, BLK, HER4 and EGFR were less affected (Table 6). As expected, no inhibition of JAK3, MKK7 and ITK was observed, which may be attributed to their bulkier gatekeeper incapable of forming the predicted hydrogen bond to the inverted amide moiety. In comparison, we observed that compound **12c** had a slightly better potency than **12a**, yet both shared a similar selectivity profile. Moreover, saturated analog **12b** expectedly showed a much weaker BMX inhibition with an IC_50_ value of 582 nM while being devoid any significant activity on other kinases in this set except BTK and TXK (70% and 89% residual activity at 200 nM, respectively). For a better selectivity ranking, we determined IC_50_ values of **12a** and **12c** against all kinases that were significantly inhibited at 200 nM (Table 6). The results confirmed that both compounds were highly potent inhibitors of BMX demonstrated by IC_50_ values of 2 nM and 1 nM, respectively. However, these inhibitors also inhibited BTK with similar potencies, suggesting that the moderate selectivity against BTK observed earlier for the parent compounds **9a** and **10a** was unfortunately compromised. Nevertheless, moderate selectivity of **12a** and **12c** over the other kinases in this test set, which included TXK, TEC, EGFR, HER2, HER4 and BLK, was evident and both had a slightly different selectivity profile. In comparison to **12c**, which exhibited 8−30-fold lower potencies for the tested kinases (>100-fold only for HER2), compound **12a** showed a better selectivity against HER family kinases (72-, 280- and 48-fold against EGFR, HER2 and HER4, respectively), TEC (14-fold) and BLK (24-fold), while selectivity against TXK (6-fold) remained moderate.

**Figure 7:**
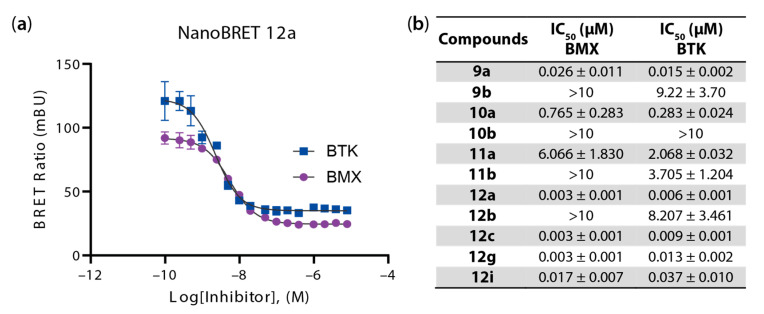
(**a**) Exemplified dose**–**response curve of **12a** titrated versus BTK (blue squares) or BMX (magenta circles) in a NanoBRET^™^ assay using HEK293T cells. Each point represents the average of triplicates with standard deviation. For dose response curves of the other inhibitors tested, see the supporting information. (**b**) IC_50_ values determined in NanoBRET^™^ assays in HEK293T cells.

Cellular target engagement was evaluated by a bioluminescence resonance energy transfer (NanoBRET™) assay [28] using HEK293T cells (see Figure 7). Lead compounds **12a** and **12c** showed favorable low nanomolar IC_50_ values against both, BMX and BTK with a slight bias towards BMX. High potency was also observed for compounds **12g,i** and early hit compound **9a** while the close analog **10a** showed decreased potency, which might be a result of lower cell penetration due to the primary amide functionality. As expected, and in line with the data from enzymatic assays, *N*-alkylated analog **11a** and non-reactive control compounds **9b**, **10b**, **11b** and **12b** showed weak or no affinity for both kinases in this cellular model.

### 2.4. Validation of Covalent Modification and Binding Mode Prediction

**Figure 8:**
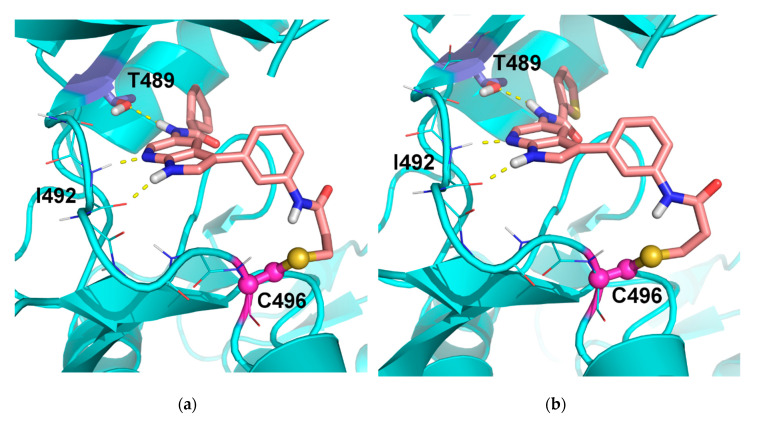
Covalent docking models of compounds **12a** (panel (**a**)) and **12c** (panel (**b**)). Poses were generated using the BMX crystal structure with the PDB-code 3SXR as the template.

Covalent adduction between BMX and the inhibitors was investigated by mass spectrometry (see Supplementary Table S1). When incubating highly potent acrylamide-derived inhibitors **9a**, **10a** and **12a**,**c**,**g**,**i** at 1.5-fold molar excess with BMX at 4 °C, complete labeling was observed readily after 30 min indicating a high efficiency of covalent bond formation. In contrast, analog **11a**, which was less potent in the activity assay required prolonged exposure (240 min) to approach full target modification. As expected, no modification was detectable for propionamides **9b**, **10b**, **11b** and **12b**, which were employed as negative controls. To examine general reactivity towards thiols, compound **12c** was tested for its stability against glutathione (GSH). In the presence of 5 mM GSH at pH 7.4, **12c** showed a half-life of approx. 10 h which compares favorably to Afatinib (<1 h) tested under the same conditions (see Supplementary Figure S2). It can therefore be concluded that covalent target modification is facilitated by the preceding reversible binding event and not a result of nonspecific thiol addition. To gain a detailed insight in binding interactions, we aimed to crystallize the complex of BMX and the inhibitors, yet unsuccessfully. Thus, covalent docking analyses were instead performed. Suggested covalent binding modes of the two representative compounds **12a** and **12c** are depicted in Figure 8. As expected, the docking poses show the dual hinge interaction pattern along with an additional hydrogen bond between the amide NH and the hydroxy group of T489 orienting the nonpolar substituent at the 5-acylamino group towards the hydrophobic region I.

Although we did not experimentally prove selective modification of C496 by tryptic digestion/MS experiments, crystal structure or mutation of the target cysteine, the efficiency and stoichiometry of covalent modification in conjunction with modeling data and the binding modes of analogs **9a** and **10a** in JAK3 strongly support the specific labeling of this residue, which is the only accessible cysteine in or proximal to the BMX ATP binding site. In addition, much lower activity of non-reactive analog **12b** compared to acrylamide **12a** on all enzymes tested complies with a covalent mode of action on other kinases with an equivalent cysteine placement.

### 2.5. Compound Synthesis

**Scheme 1:**
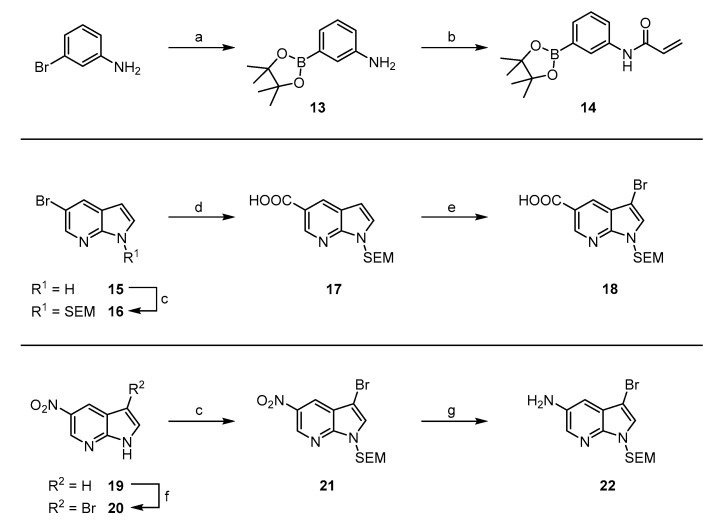
Synthesis of key building blocks: (a) B_2_pin_2_, XPhos Pd G4, KOAc, dioxane, 90 °C (84%); (b) acryloyl chloride, Et_3_N, DCM, −10°C (85%); (c) NaH, SEM-Cl, DMF, 0°C to rt (56–78%) (d) *n*-BuLi, CO_2_, THF. −78 °C (51%); (e) Br_2_, DCM, 0°C (89%); (f) NBS, DMF, rt (70%); (g) Zn^0^, ammonium formate, EtOH, 50 °C (51%).

The key transformation in the synthetic route to the inhibitors disclosed herein was the late stage introduction of the entire *N-*aryl acrylamide warhead/linker fragment via a Suzuki coupling. This reaction could be performed under very mild conditions preventing possible side reactions involving the electrophilic Michael acceptor system. The synthesis of the key boronic acid ester **14** was realized as a high yielding two step sequence starting from 3-bromoaniline. The latter was smoothly converted to the pinacol boronate **13** under Miyaura conditions [35] followed by the acylation with acryloyl chloride to deliver the bench stable building block **14** in a good overall yield of 71% (Scheme 1, top).

For the facile derivatization in position 5 of the 7-azaindole scaffold, the corresponding 5-amino and 5-carboxy functionalized key intermediates **18** and **22** were prepared. The synthesis of **18** started with the SEM-protection of commercially available 5-bromo-7-azaindole (**15**) to deliver compound **16**. The carboxy group was then introduced via lithium–halogen-exchange followed by quenching the intermediate aryllithium species with gaseous carbon dioxide at low temperatures. The treatment with elemental bromine in methylene chloride converted acid **17** cleanly to the desired intermediate **18** in excellent yields (Scheme 1, middle).

The corresponding 5-amino derivative **22** was prepared from 5-nitro-7-azaindole **19**, which is accessible via a previously reported scalable three-step process [36]. Starting from intermediate **19**, the 3-bromo substituent was introduced via electrophilic bromination with NBS (**20**) followed by the SEM-protection of the indole nitrogen atom (**21**). Finally, the nitro group was reduced under mild conditions with zinc and ammonium formate to obtain the key building block **22** (Scheme 1, bottom).

**Scheme 2:**
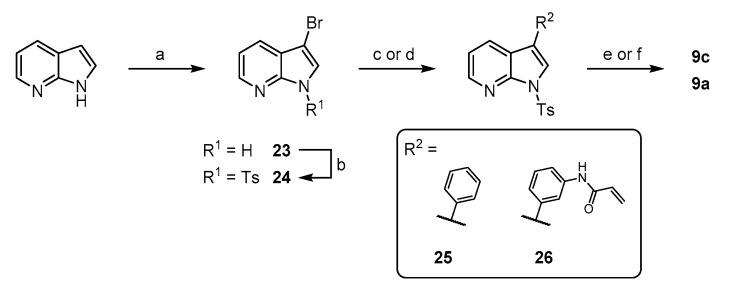
Synthesis of compounds **9c** and **9a**. (a) NBS, DMF, rt (96%); (b) NaH, TsCl, THF, 0 °C to rt (85%); (c) phenylboronic acid, Pd(OAc)_2_, XPhos, Na_2_CO_3_, dioxane/H_2_O, 70 °C (82%); (d) **14**, XPhos Pd G3, K_2_CO_3_, dioxane/H_2_O, 50 °C (quant.); (e) for **9c**: KOH, MeOH, 60°C (85%); (f) for **9a**: KOH, *t*BuOH, 50 °C (81%).

**Scheme 3:**
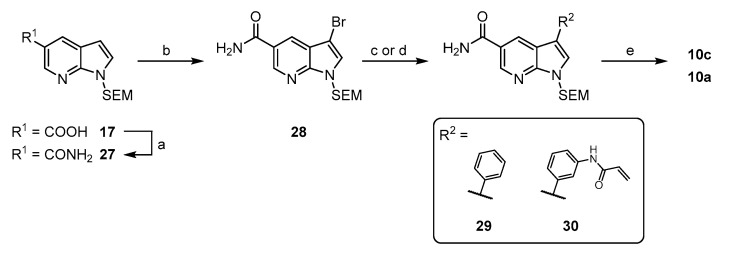
Synthesis of compounds **10c** and **10a**. (a) CDI, NH_3(aq)_, DMF, rt (72%); (b) Br_2_, DCM, 0 °C (68%); (c) phenylboronic acid, *t*Bu_3_P Pd G3, K_3_PO_4_, dioxane/H_2_O, rt (quant.); (d) **14**, *t*Bu_3_P Pd G3, K_2_CO_3_, dioxane/H_2_O, rt (93%); (e) TFA, DCM, rt (71–92%).

The early hit compounds **9a** and **10a** and their corresponding unsubstituted phenyl analogs **9c** and **10c** were synthesized following slightly modified routes (Scheme 2 and Scheme 3). Starting from plain 7-azaindole, the bromination of position 3 was performed with NBS to give compound **23** in almost quantitative yield. In the following step, the indole nitrogen atom was protected with a tosyl group via deprotonation with sodium hydride and reaction with tosyl chloride to obtain intermediate **24**. The latter was coupled with phenylboronic acid or building block **14** under Suzuki conditions to yield the compounds **25** and **26**, respectively. Tosyl deprotection under basic conditions in methanol or *tert*-butanol, respectively, delivered the final compounds **9c** and **9a**.

The corresponding derivatives with an unsubstituted carboxamide in position 5 (**10c** and **10a**) were accessible from the SEM-protected acid **17**. CDI-mediated coupling of the latter with ammonia led to carboxamide **27**. Bromination in position 3 with elemental bromine afforded intermediate **28**, which was then Suzuki-coupled with phenylboronic acid or building block **14**, respectively. The precursors **29** and **30** were finally deprotected under acidic conditions with TFA in DCM to obtain compounds **10c** and **10a** (Scheme 3).

**Scheme 4:**
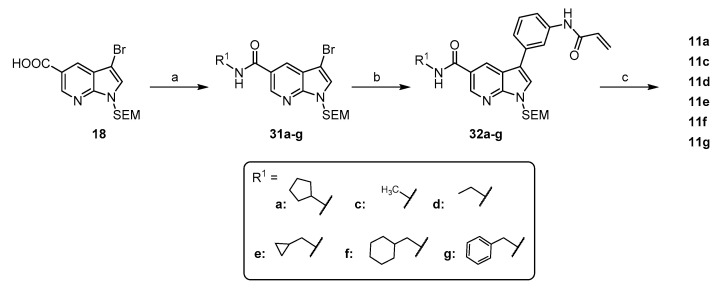
Synthesis of *N-*alkylated 7-azaindole-5-carboxamides. (a) CDI, corresponding amine, DMF, rt (46–80%); (b) **14**, *t*Bu_3_P Pd G3, K_3_PO_4_ or K_2_CO_3_, dioxane/H_2_O, rt (53–79%); (c) TFA, DCM, rt (23–97%).

The series of *N*-substituted azaindole 5-carboxamides (compounds **11a** and **11c**–**g**) was accessible via a three-step procedure starting from key intermediate **18** (Scheme 4). First, amides **31**a–**g** were prepared via CDI-mediated acid activation and reaction with the corresponding amines. The latter intermediates were then coupled with **14** under mild Suzuki conditions at ambient temperature to give **32a**–**g** in favorable yields. The final compounds **11a** and **11c**–**g** were obtained after acid-promoted SEM-cleavage using TFA in DCM at ambient temperature (Scheme 4).

**Scheme 5:**
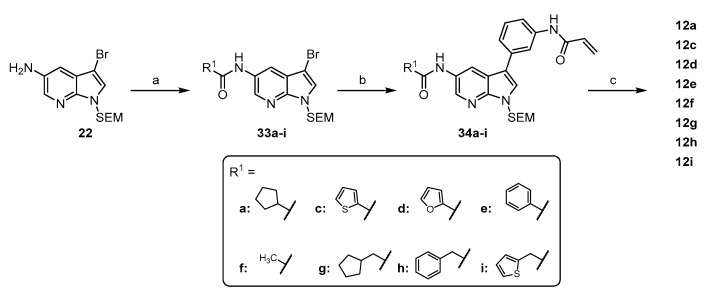
Synthesis of 5-acylamino-7-azaindoles. (a) corresponding acid chloride, Et_3_N, DCM, rt (47–90%); (b) **14**, *t*Bu_3_P Pd G3, K_3_PO_4_ or K_2_CO_3_, dioxane/H_2_O, rt (45–73%); (c) TFA, DCM, rt (29–77%).

The final inhibitor series **12a** and **12c–i** featuring an inverted amide functionality was prepared via a similar route as described for **11a** and **11c–g** (Scheme 5). Building block **22** was reacted with the corresponding acid chlorides to deliver the amide derivatives **33**a–**i** in moderate to excellent yields. The Suzuki coupling with boronate ester **14** was performed under the mild conditions established before to afford precursors **34a**–**i**. The latter were converted to the final compounds **12a** and **12**c–**i** under the same acidic conditions as described above (Scheme 5).

**Scheme 6:**
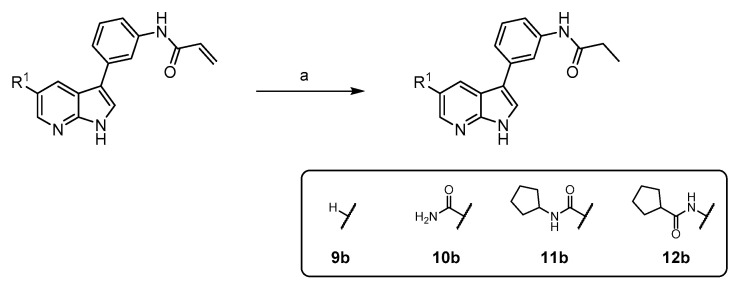
Synthesis of propionamides **9b**, **10b**, **11b** and **12b** as negative controls. (a) H_2_ (1 bar), Pd/C, THF/MeOH, (60–95%).

To access the propionamide analogs of selected inhibitors as negative control compounds, the corresponding acrylamides were hydrogenated in the presence of a Pd/C catalyst. Due to the absence of sensitive functional groups, these conversions usually proceeded smoothly and gave the desired propionamides **9b**, **10b**, **11b** and **12b** in good to excellent yields (Scheme 6).

## 3. Discussion

Here we describe the discovery of a novel class of covalent inhibitors of the TEC family kinases, most notably BMX and BTK. Development started from compounds **9a** and **10a** belonging to an unprecedented class of covalent JAK3 inhibitors, which possessed potent off-target activity on the TEC kinases BMX and TXK while showing moderate to high selectivity against the other eight protein kinases with an equivalent cysteine placement. Guided by the crystal structures of **9a** and **10a** in complex with JAK3, modification of the hinge binding motif as well as back pocket binding moieties was successfully exploited as a strategy to fine tune the binding profiles of these irreversible inhibitors that target a cysteine located at the αD-1 position. This approach enabled the development of irreversible inhibitors for BMX and BTK, which showed a general preference for kinases with a less bulky threonine gatekeeper residue, enabling high selectivity over JAK3 and MKK7 that harbor a methionine, and ITK that possess a phenylalanine at this position.

The key step in the synthetic access to these inhibitors was the late stage introduction of the *N*-phenyl acrylamide warhead via Suzuki coupling under exceptionally mild conditions. This facilitated broad SAR evaluation and fast library synthesis. Starting from unsubstituted carboxamide **10a**, we introduced *N*-alkyl substituents to induce a clash with bulkier gatekeeper moieties, but this modification led to a drop in potency on both JAK3 and BMX. In contrast, inversion of the carboxamide in the 5-position of the azaindole scaffold retained BMX inhibitory potency, while activity on JAK3 was strongly decreased. Besides the expected steric clash with the methionine gatekeeper moiety in JAK3, molecular modeling suggested inverted amides to form an additional hydrogen bond towards the hydroxy group of threonine gatekeeper moieties, which drives the carbonyl’s substituent towards the hydrophobic region I.

The most promising compounds **12a** and **12c** were further profiled against the TEC kinase family and other kinases with an αD-1 cysteine. As expected, low activity was detected on kinases with a non-threonine gatekeeper. However, the promising selectivity pattern observed for starting compounds **9a** and **10a** was partially lost. Most notably, optimization was accompanied by a strong increase in BTK potency with compound **12a** being equipotent on BMX and BTK, while **12c** slightly favored BTK with subnanomolar inhibitory activity in the enzymatic assay. Moderate selectivity was observed against the other TEC kinases and BLK. However, compound **12c** also showed significant off-target activity on HER family kinases while **12a** maintained good selectivity against the latter. Comparison with unreactive analog **12b**, which showed weak activity on all tested kinases underlined the importance of the covalent mode of action. Highly efficient covalent modification of BMX was experimentally shown for several analogs via mass spectrometry while potent cellular BMX and BTK engagement was demonstrated by means of a NanoBRET^TM^ assay.

Targeting BMX has been proposed as a strategy for treatment of various diseases including cardiovascular disorders [37] and certain cancers [26,38,39]. Nevertheless, only three inhibitors have been developed to date: BMX-IN-1 (**6**, see Figure 1), the analog JS25 (**7a**) and the type II inhibitor CHMFL-BMX-078 (**8**). However, these inhibitors still exhibit pronounced affinities to several other kinases with different selectivity profiles. Off-target activities could thus limit their use as a sole tool for biological study. Moreover, their chemical structures suggest unfavorable physicochemical properties due to size and the high portion of sp^2^ atoms. The dual covalent BMX/BTK inhibitors presented here thus complement the repository of available BMX inhibitors. It should be mentioned, however, that caution must be taken when comparing covalent inhibitors based on (apparent) IC_50_ data since the latter depend on incubation time. For example, it has recently been demonstrated in the case of MKK7 and ibrutinib that a longer incubation could result in a misled nanomolar inhibitory activity observed for an inhibitor that has only weak reversible binding affinity to the kinase [16]. Nonetheless, determination of meaningful kinetic data, i.e., *k*_inact_ and *K*_I_, to disentangle contributions of reversible binding and the subsequent bond-forming step remains highly elaborate, thus for BMX such values have only been reported for JS25 and a few related compounds from the same study [27]. Moreover, and in contrast to the aforementioned studies, the compounds presented herein have not been characterized in terms of wider kinome selectivity. Improvements also need to be made concerning the intra-TEC family selectivity. Future efforts will primarily focus on increasing the selectivity against BTK, for example by modifying the residues targeting the hydrophobic region I or by extending the latter towards the DFG-out pocket to generate type II inhibitors [16].

Overall, the presented optimization approach offers a strategy that can be exploited to fine tune existing covalent inhibitors towards unrelated kinases. Compared to previous compounds, the BMX inhibitors discovered here are based on an alternative and readily optimizable chemotype, which may be beneficial for further development of probes that exclusively target BMX. Currently, there is no selective inhibitor or chemical probe available for BMX. However, combinatorial use of our dual irreversible BMX/BTK inhibitors along with other inhibitors with different off-target profiles as a set of chemogenomic compounds would nevertheless allow dissecting biological functions of BMX as well as its role in disease development, which will enable further validation of this kinase as a potential therapeutic target.

## 4. Materials and Methods

### 4.1. Chemical Synthesis

#### 4.1.1. General Information

Chemical synthesis was carried out using commonly applied techniques and general procedures. All starting materials and reagents were of commercial quality and were used without further purification. Thin layer chromatography (TLC) was carried out on Merck 60 F254 silica plates (Merck KGaA, Darmstadt, Germany) and were visualized under UV light (254 nm and 366 nm) or developed with an appropriate staining reagent. Preparative column chromatography was carried out with an Interchim PuriFlash 430 or PuriFlash XS420 (Interchim S.A., Montlucon, Allier, France) automated flash chromatography system on normal phase silica gel (Grace Davison Davisil LC60A 20–45 micron (W.R. Grace and Company, Columbia, MD, USA) or Merck Geduran Si60 63–200-micron silica (Merck KGaA, Darmstadt, Germany).

Nuclear magnetic resonance (NMR) spectral analysis was performed on Bruker Avance 200 or Bruker Avance 400 instruments (Bruker Corporation, Billerica, MA, USA). The samples were dissolved in deuterated solvents and chemical shifts are given in relation to tetramethylsilane (TMS). Spectra were calibrated using the residual peaks of the used solvent.

Mass spectrometry (MS) was carried out with an Advion TLC-MS interface (Advion, Ithaca, NY, USA) with electron spray ionization (ESI) in positive and/or negative mode. Instrument settings were as follows: ESI voltage 3.50 kV, capillary voltage 187 V, source voltage 44 V, capillary temperature 250 °C, desolvation gas temperature 250 °C, gas flow 5 L/min nitrogen.

Purities of final compounds were determined via high performance liquid chromatography (HPLC) using an Agilent 1100 Series LC system (Agilent Technologies, Santa Clara, CA, USA) with Phenomenex Luna C8 columns (150 × 4.6 mm, 5 µm) (Phenomenex Inc. Torrance, CA, USA) and detection was performed with a UV DAD at 254 nm and 230 nm wavelength. Elution was carried out with the following gradient: 0.01 M KH_2_PO_4_, pH 2.30 (solvent A), MeOH (solvent B), 40% B to 85% B in 8 min, 85% B for 5 min, 85% to 40% B in 1 min, 40% B for 2 min, stop time 16 min, flow 1.5 mL/min. All final compounds showed a purity above 95% in the means of peak area at the two different wavelengths.

#### 4.1.2. General Synthetic Procedures

*General Procedure A (Suzuki coupling):* In a screw-top reaction vial were combined the corresponding boronic acid or ester and the aryl bromide under argon atmosphere. Dioxane and the aqueous base were added subsequently, and the mixture was degassed carefully via several vacuum/argon cycles. Finally, the catalyst system was added to the reaction and the degassing procedure was repeated. The vial was sealed and heated to the indicated temperature until reaction control (TLC or HPLC) showed complete consumption of starting materials. The reaction was cooled to ambient temperature, diluted with EtOAc and washed with brine. The organic phase was dried over Na_2_SO_4_ and evaporated to dryness. The residue was usually purified via flash chromatography using an appropriate solvent system.

*General Procedure B (SEM cleavage)*: The SEM-protected substrate was dissolved in dry DCM and TFA was added subsequently. The reaction was stirred until TLC indicated complete consumption of the starting material. The majority of TFA was removed under reduced pressure and the residue was taken up in EtOH and was basified with aqueous NH_3_ solution. The mixture was stirred at ambient temperature until complete consumption of the hydroxymethyl intermediate (usually overnight) was observed. The volatiles were stripped of under reduced pressure and the residue purified via flash chromatography as indicated.

*General Procedure C (amide coupling with CDI)*: Carboxylic acid **18** was dissolved in dry DMF (ca. 0.1 M) and CDI was added at ambient temperature. The mixture was stirred until gas evolution ceased (usually about 1 h) before the corresponding amine was added to the reaction. Stirring was continued until reaction control indicated complete conversion and the reaction was quenched by addition of water followed by dilution with EtOAc. The organic phase was washed with sat. NaHCO_3_ (two times) and brine, prior to solvent evaporation and purification by flash chromatography.

*General Procedure D (amide coupling with acid chlorides):* To a solution of **22** and Et_3_N in dry DCM (ca. 0.1 M) was added the corresponding acid chloride under ice-cooling. After complete addition, the cooling bath was removed and stirring was continued at ambient temperature until reaction control (HPLC or TLC) indicated complete conversion. The reaction was quenched by addition of water followed by dilution with EtOAc. The organic phase was washed with sat. NaHCO_3_, and brine, prior to drying over Na_2_SO_4_ and evaporation. The residue was purified via flash chromatography with an appropriate eluent system.

#### 4.1.3. Synthesis and Characterization of Intermediates and Final Compounds

*3-(4,4,5,5-tetramethyl-1,3,2-dioxaborolan-2-yl)aniline* (**13**): In a flame-dried Schlenk flask were suspended 1.47 g dry KOAc (15.0 mmol), 860 mg 3-bromoaniline (5.00 mmol) and 1.27 g bis(pinacolato)diboron (5.00 mmol) in 20 mL dry dioxane. The mixture was carefully degassed via three vacuum/argon cycles and 9 mg XPhos Pd G4 (10 µmol) was added subsequently. The degassing procedure was repeated and then the reaction was heated to 90 °C oil-bath temperature overnight. After cooling to ambient temperature, the mixture was diluted with EtOAc and filtered over a bed of celite. The filtrate was washed with water (three times) and brine, prior to drying over Na_2_SO_4_ and evaporation. The residue was triturated with heptane and the precipitate isolated by filtration to yield 914 mg (84%) of the product as a slightly brownish solid. ^1^H NMR (200 MHz, DMSO) δ 7.06–6.97 (m, 1H), 6.95 (d, *J* = 1.9 Hz, 1H), 6.82 (dt, *J* = 7.2, 1.1 Hz, 1H), 6.66 (ddd, *J* = 7.9, 2.4, 1.1 Hz, 1H), 5.02 (br s, 2H), 1.26 (s, 12H) ^13^C NMR (50 MHz, DMSO) δ 148.0, 128.3, 128.3, 121.9, 120.1, 116.8, 83.2, 24.7 TLC-MS (ESI) *m*/*z*: 219.9 [M + H]^+^ HPLC t_ret_ = 6.97 min.

*N-(3-(4,4,5,5-tetramethyl-1,3,2-dioxaborolan-2-yl)phenyl)acrylamide* (**14**): To a solution of 891 mg **13** (4.07 mmol) and 715 µL Et_3_N (5.09 mmol) in 16 mL dry DCM were dropwise added 368 µL acryloyl chloride (4.48 mmol) at −10 °C. After 1 h, the reaction was quenched by addition of sat. NH_4_Cl and transferred to a separatory funnel. The phases were separated and the organic phase was washed with sat NaHCO_3_ and brine prior to drying over Na_2_SO_4_ and evaporation. The crude product was purified via flash chromatography (petrol ether/EtOAc + 5% MeOH (10–40%)) to obtain 780 mg (85%) of the title compound as a white solid. ^1^H NMR (200 MHz, DMSO) δ 10.14 (br s, 1H), 7.98 (d, *J* = 1.4 Hz, 1H), 7.85 (dt, *J* = 6.3, 2.4 Hz, 1H), 7.41–7.26 (m, 2H), 6.43 (dd, *J* = 17.0, 9.7 Hz, 1H), 6.25 (dd, *J* = 17.0, 2.4 Hz, 1H), 5.75 (dd, *J* = 9.7, 2.4 Hz, 1H), 1.29 (s, 12H) ^13^C NMR (50 MHz, CDCl_3_) δ 163.1, 138.6, 131.8, 129.3, 128.4, 126.8, 125.3, 122.2, 83.7, 24.7 TLC-MS (ESI) *m*/*z*: 296.4 [M + H]^+^ HPLC t_ret_ = 7.45 min.

*5-bromo-1-((2-(trimethylsilyl)ethoxy)methyl)-1H-pyrrolo[2,3-b]pyridine* (**16**): To an ice-cooled solution of 1.96 g 5-bromo-1*H*-pyrrolo[2,3-*b*]pyridine (10 mmol) in 10 mL dry DMF were added 518 mg sodium hydride (60%wt dispersion in min. oil, 12.9 mmol) and stirring was continued for about 30 min. Subsequently were added 1.95 mL SEM-Cl (10.9 mmol) and stirring was continued until TLC indicated complete conversion. The reaction was diluted with 120 mL EtOAc and transferred to a separatory funnel. The organic phase was successively washed with water (four times) and brine, prior to drying over Na_2_SO_4_ and evaporation. The residue was purified via flash chromatography (petrol ether/EtOAc (0–15%)) to obtain 2.55 g (78%) as a colorless oil. ^1^H NMR (200 MHz, CDCl_3_) δ 8.34 (d, *J* = 1.9 Hz, 1H), 8.02 (d, *J* = 1.9 Hz, 1H), 7.35 (d, *J* = 3.6 Hz, 1H), 6.46 (d, *J* = 3.6 Hz, 1H), 5.64 (s, 2H), 3.52 (t, *J* = 8.2 Hz, 2H), 0.89 (t, *J* = 8.2 Hz, 2H), −0.07 (s, 9H) ^13^C NMR (50 MHz, CDCl_3_) δ 146.6, 143.8, 131.1, 129.5, 122.3, 112.3, 100.7, 73.2, 66.5, 17.9, −1.3 TLC-MS (ESI) *m*/*z*: 349.3 [M + Na]^+^ HPLC t_ret_ = 10.84 min.

*1-((2-(trimethylsilyl)ethoxy)methyl)-1H-pyrrolo[2,3-b]pyridine-5-carboxylic acid* (**17**): Under argon atmosphere were dissolved 2.49 g **16** (7.6 mmol) in 80 mL dry THF in a flame-dried Schlenk tube. At –78 °C, 3.3 mL of a 2.5 M *n*-BuLi solution in hexane (8.36 mmol) was added dropwise. After complete addition, stirring was continued for 30 min before dry CO_2_ gas was bubbled through the solution. After another 30 min, the reaction was quenched with water and slowly warmed to ambient temperature. After dilution with EtOAc, the organic phase was extracted with 0.5 M aqueous NaOH (three times). The combined extracts were acidified with 2M HCl and back-extracted with EtOAc (three times). The combined organic phases were washed with brine, dried over Na_2_SO_4_ and evaporated to dryness. The crude product was purified via flash chromatography (petrol ether/EtOAc + 5% MeOH + 2% AcOH (25–75%)) to yield 1.14 g (51%) of pure **17** as an off-white semisolid. ^1^H NMR (200 MHz, CDCl_3_) δ 10.39 (br s, 1H), 9.10 (s, 1H), 8.65 (s, 1H), 7.43 (d, *J* = 3.5 Hz, 1H), 6.64 (d, *J* = 3.5 Hz, 1H), 5.73 (s, 2H), 3.55 (t, *J* = 8.2 Hz, 2H), 0.90 (t, *J* = 8.2 Hz, 2H), -0.09 (s, 9H) ^13^C NMR (50 MHz, CDCl_3_) δ 171.4, 150.0, 145.8, 132.0, 129.8, 120.3, 118.7, 102.8, 73.3, 66.6, 17.8, −1.4 TLC-MS (ESI) *m*/*z*: 291.4 [M − H]^−^ HPLC t_ret_ = 8.66 min.

*3-bromo-1-((2-(trimethylsilyl)ethoxy)methyl)-1H-pyrrolo[2,3-b]pyridine-5-carboxylic acid* (**18**): In a 100 mL round-bottomed flask were dissolved 1.14 g **17** (3.89 mmol) in 50 mL dry DCM and the solution was cooled in an ice/water bath. To this were added 684 mg bromine (4.28 mmol) as 0.5 M solution in DCM in small portions until the persistence of yellow coloration was observed. The reaction was quenched by addition of aqueous Na_2_S_2_O_3_ solution and extracted with DCM (three times). The combined extracts were washed with sat. NH_4_Cl, water and brine, prior to drying over Na_2_SO_4_ and evaporation. The residue was subjected to flash purification (petrol ether/EtOAc + 5% MeOH + 2% AcOH (25–75%)) to obtain 1.29 g (89%) of **18** as a brownish oil. ^1^H NMR (200 MHz, CDCl_3_) δ 11.16 (br s, 1H), 9.10 (s, 1H), 8.59 (s, 1H), 7.48 (s, 1H), 5.70 (s, 2H), 3.56 (t, *J* = 8.2 Hz, 2H), 0.92 (t, *J* = 8.2 Hz, 2H), −0.07 (s, 9H) ^13^C NMR (50 MHz, CDCl_3_) δ δ 171.1, 149.1, 146.8, 130.9, 128.7, 119.8, 119.3, 91.8, 73.2, 66.9, 17.8, −1.4 TLC-MS (ESI) *m*/*z*: 369.2 [M − H]^−^ HPLC t_ret_ = 9.82 min.

*3-bromo-5-nitro-1H-pyrrolo[2,3-b]pyridine* (**20**): To an ice-cooled suspension of 1.8 g 5-nitro-1*H*-pyrrolo[2,3-*b*]pyridine (11.0 mmol) in 40 mL dry DMF were added 2.36 g *N*-bromosuccinimide (13.2 mmol) as solid in small portions. After complete addition, the cooling bath was removed and the reaction was stirred for 3 h at ambient temperature. The mixture was diluted with water and aqueous Na_2_S_2_O_3_ and the precipitate was isolate by filtration. The filter cake was washed with water and dried at 60 °C in a convection oven to yield 1.86 g (70%) of the title compound as a yellow solid. ^1^H NMR (200 MHz, DMSO-*d*_6_) δ 12.95 (s, 1H), 9.18 (d, *J* = 2.5 Hz, 1H), 8.64 (d, *J* = 2.5 Hz, 1H), 8.09 (d, *J* = 2.6 Hz, 1H). TLC-MS (ESI) *m*/*z*: 239.1 [M − H]^−^ HPLC t_ret_ = 7.73 min.

*3-bromo-5-nitro-1-((2-(trimethylsilyl)ethoxy)methyl)-1H-pyrrolo[2,3-b]pyridine* (**21**): To an ice-cooled solution of 2.14 g **20** (8.9 mmol) in 20 mL dry DMF were added 462 mg sodium hydride (60%wt dispersion in min. oil, 11.5 mmol) and stirring was continued for about one h. Subsequently were added 1.7 mL SEM-Cl (9.7 mmol) and stirring was continued until TLC indicated complete conversion. The reaction was diluted with 120 mL EtOAc and transferred to a separatory funnel. The organic phase was successively washed with water (four times) and brine, prior to drying over Na_2_SO_4_ and evaporation. The residue was purified via flash chromatography (petrol ether/EtOAc (0–20%)) to obtain 1.85 g (56%) **21** as a yellow oil. ^1^H NMR (200 MHz, CDCl_3_) δ 9.24 (d, *J* = 2.4 Hz, 1H), 8.73 (d, *J* = 2.4 Hz, 1H), 7.58 (s, 1H), 5.70 (s, 2H), 3.54 (m, 2H), 0.92 (m, 2H), −0.05 (s, 9H). HPLC t_ret_ = 11.56 min.

*3-bromo-1-((2-(trimethylsilyl)ethoxy)methyl)-1H-pyrrolo[2,3-b]pyridin-5-amine* (**22**): To a solution of 1.85 g **21** (5.0 mmol) in 40 mL abs. EtOH were added 1.63 g finely powdered zinc (24.9 mmol) and 1.57 g ammonium formate (24.9 mmol). The mixture was stirred for 22 h at 50 °C and was then filtered over a celite pad to remove unreacted zinc. The filtrate was concentrated under reduced pressure and taken up in EtOAc. The organic phase was washed with sat NH_4_Cl and brine, prior to drying over Na_2_SO_4_ and evaporation. The residue was purified via flash chromatography (petrol ether/EtOAc 20–60%) to obtain 0.86 g (51%) of the title compound as a brown oil. ^1^H NMR (200 MHz, CDCl_3_) δ 7.93 (d, *J* = 2.6 Hz, 1H), 7.27 (s, 1H), 7.14 (d, *J* = 2.6 Hz, 1H), 5.55 (s, 2H), 4.60 (s, 2H), 3.51 (t, 2H), 0.88 (t, 2H), −0.08 (s, 9H). ^13^C NMR (50 MHz, CDCl_3_) δ 142.1, 137.5, 134.6, 127.2, 120.2, 112.5, 88.5, 72.9, 66.2, 17.7, −1.6. HPLC t_ret_ = 8.65 min.

*3-bromo-1H-pyrrolo[2,3-b]pyridine* (**23**): To an ice-cooled solution of 473 mg 1*H*-pyrrolo[2,3-*b*]pyridine (4.0 mmol) in 8 mL DMF were added 726 mg *N*-bromosuccinimide (4.1 mmol) in several portions. After complete addition, the cooling bath was removed and stirring was continued for 4 h. The reaction mixture was poured on sat. NaHCO_3/_ice and the resulting suspension was stirred for ca. 10 min until a homogenous precipitate was formed. The solids were collected by filtration, washed with water and dried in vacuo to yield 753 mg (96%) of the title compound as a white solid. ^1^H NMR (200 MHz, CDCl_3_) δ 12.07 (br s, 1H), 8.29 (dd, *J* = 4.7, 1.5 Hz, 1H), 7.83 (dd, *J* = 7.9, 1.5 Hz, 1H), 7.70 (s, 1H), 7.16 (dd, *J* = 7.9, 4.7 Hz, 1H) ^13^C NMR (50 MHz, DMSO) δ 147.2, 143.9, 126.4, 125.6, 118.7, 116.3, 87.1 TLC-MS (ESI) *m*/*z*: 197.1 [M + H]^+^ HPLC t_ret_ = 6.56 min.

*3-bromo-1-tosyl-1H-pyrrolo[2,3-b]pyridine* (**24**): To an ice-cooled solution of 197 mg **23** (1.0 mmol) in 5 mL dry THF were added 50 mg sodium hydride (60%wt dispersion in min. oil, 1.25 mmol) and stirring was continued for about 30 min. Subsequently were added 210 mg TsCl (1.1 mmol) and stirring was continued until TLC indicated complete conversion. The reaction was diluted with 25 mL EtOAc and transferred to a separatory funnel. The organic phase was successively washed with water and brine, prior to drying over Na_2_SO_4_ and evaporation. The residue was triturated with chilled MeOH and filtered to obtain 298 mg (85%) **24** as a white solid. ^1^H NMR (200 MHz, DMSO) δ 8.49–8.40 (m, 1H), 8.20 (s, 1H), 8.01 (d, *J* = 8.4 Hz, 2H), 7.96–7.86 (m, 1H), 7.46–7.33 (m, 3H), 2.31 (s, 3H) ^13^C NMR (50 MHz, DMSO) δ 146.1, 146.0, 145.4, 134.2, 130.2, 128.7, 127.8, 125.7, 121.7, 120.2, 95.1, 21.1 HPLC t_ret_ = 8.86 min.

*3-phenyl-1-tosyl-1H-pyrrolo[2,3-b]pyridine* (**25**): The preparation was performed following *General Procedure A* from 100 mg **24** (0.25 mmol) and 38 mg phenylboronic acid (0.31 mmol), catalyzed by 1 mg Pd(OAc)_2_ (5 µmol) and 5 mg XPhos (10 µmol) in 3 mL dioxane and 0.75 mL of an 1 M Na_2_CO_3_ solution. The reaction was conducted at 70 °C and the crude product was purified via flash chromatography (petrol ether/EtOAc + 5% MeOH (5–30%)) to obtain 72 mg (82%) of the title compound as a colorless semisolid. ^1^H NMR (200 MHz, CDCl_3_) δ 8.50 (dd, *J* = 4.7, 1.2 Hz, 1H), 8.25–8.04 (m, 3H), 7.92 (s, 1H), 7.68–7.56 (m, 2H), 7.55–7.17 (m, 6H), 2.36 (s, 3H) ^13^C NMR (50 MHz, CDCl_3_) δ 147.6, 145.3, 145.1, 135.4, 132.6, 129.7, 129.1, 128.9, 128.1, 127.7, 127.4, 122.7, 121.6, 120.3, 119.1, 21.6 TLC-MS (ESI) *m*/*z*: 403.0 [M + Na + MeOH]^+^ HPLC t_ret_ = 9.02 min.

*N-(3-(1-tosyl-1H-pyrrolo[2,3-b]pyridin-3-yl)phenyl)acrylamide* (**26**): The preparation was performed following *General Procedure A* from 53 mg **24** (0.15 mmol) and 51 mg **14** (0.19 mmol), catalyzed by 3 mg XPhos Pd G3 (5 µmol) in 3.6 mL dioxane and 0.9 mL of an 0.5 M K_2_CO_3_ solution. The reaction was conducted at 50 °C and the crude product was purified via flash chromatography (petrol ether/EtOAc + 5% MeOH (40–70%)) to obtain 63 mg (quant.) of the title compound as a colorless waxy solid. ^1^H NMR (200 MHz, CDCl_3_) δ 8.47–8.35 (m, 5H), 8.23–7.95 (m, 5H), 7.85 (s, 1H), 7.57–7.45 (m, 1H), 7.40–7.11 (m, 5H), 6.52–6.24 (m, 2H), 5.74 (dd, *J* = 8.7, 2.5 Hz, 1H), 2.34 (s, 3H) ^13^C NMR (50 MHz, CDCl_3_) δ 164.3, 147.5, 145.5, 145.1, 138.8, 135.2, 133.2, 131.3, 129.8, 129.6, 129.2, 128.0, 127.9, 123.3, 122.8, 121.5, 120.1, 119.3, 119.2, 21.6 TLC-MS (ESI) *m*/*z*: 416.5 [M − H]^−^ HPLC t_ret_ = 8.00 min.

*3-phenyl-1H-pyrrolo[2,3-b]pyridine* (**9c**): In a 25 mL round-bottomed flask were dissolved 72 mg **25** (0.3 mmol) in 6 mL of a 1 M solution of KOH in MeOH at 60 °C oil-bath temperature. The reaction was stirred for 3 h and was then quenched by the addition of sat. NH_4_Cl solution. The aqueous phase was extracted with EtOAc (4 × 15 mL) and the combined extracts were washed with brine. After drying over Na_2_SO_4_ and evaporation, the residue was purified via flash chromatography (DCM/EtOAc (10–80%)) to yield 34 mg (85%) of the final compound as a white solid. ^1^H NMR (400 MHz, DMSO) δ 11.92 (br s, 1H), 8.35–8.20 (m, 2H), 7.87 (s, 1H), 7.77–7.64 (m, 2H), 7.49–7.36 (m, 2H), 7.25 (t, *J* = 7.9 Hz, 1H), 7.17–7.08 (m, 1H) ^13^C NMR (100 MHz, DMSO) δ 149.1, 142.9, 135.0, 128.8, 127.4, 126.2, 125.6, 123.6, 117.3, 116.0, 114.3 TLC-MS (ESI) *m*/*z*: 193.0 [M − H]^−^ HPLC t_ret_ = 7.60 min.

*N-(3-(1H-pyrrolo[2,3-b]pyridin-3-yl)phenyl)acrylamide* (**9a**): To a suspension of 63 mg **26** (0.15 mmol) in 10 mL *t*BuOH were added 561 mg finely powdered KOH (10 mmol) and the mixture was heated to 50 °C oil-bath temperature until TLC indicated complete conversion. The reaction was quenched by the addition of sat. NH_4_Cl solution, followed by extraction with EtOAc (3 × 20 mL). The combined extracts were washed with brine and dried over Na_2_SO_4_. After evaporation of the volatiles, the residue was purified via flash chromatography (DCM/MeOH (2–8%)) to yield 32 mg (81%) of the final compound as a white solid. ^1^H NMR (200 MHz, DMSO) δ 11.94 (s, 1H), 10.22 (s, 1H), 8.39–8.23 (m, 2H), 8.14 (br s, 1H), 7.86 (d, *J* = 2.5 Hz, 1H), 7.63–7.50 (m, 1H), 7.47–7.30 (m, 2H), 7.19 (dd, *J* = 7.9, 4.9 Hz, 1H), 6.48 (dd, *J* = 16.8, 9.7 Hz, 1H), 6.29 (dd, *J* = 16.8, 2.1 Hz, 1H), 5.78 (dd, *J* = 9.7, 2.1 Hz, 1H) ^13^C NMR (50 MHz, DMSO) δ 163.3, 149.1, 143.0, 139.5, 135.5, 132.0, 129.3, 127.4, 126.9, 123.7, 121.4, 117.2, 117.1, 116.6, 116.1, 114.1 TLC-MS (ESI) *m*/*z*: 318.5 [M + Na + MeOH]^+^ HPLC t_ret_ = 5.42 min.

*1-((2-(trimethylsilyl)ethoxy)methyl)-1H-pyrrolo[2,3-b]pyridine-5-carboxamide* (**27**): To a solution of 235 mg **17** (0.8 mmol) were added 156 mg CDI (0.96 mmol) and stirring was continued at ambient temperature for 30 min. Then 5 mL conc. NH_3_ solution were added and after another hour of stirring the reaction was further diluted with water and transferred to a separatory funnel. The aqueous phase was extracted with EtOAc (3 × 20 mL) and the combined organic phases were backwashed two times with brine. After drying over Na_2_SO_4_ and evaporation, the residue was purified via flash chromatography (petrol ether/EtOAc + 5% MeOH (40–100%)) to obtain 169 mg (72%) of the title compound as a white solid. ^1^H NMR (200 MHz, CDCl_3_) δ 8.82 (s, 1H), 8.40 (s, 1H), 7.38 (d, *J* = 3.3 Hz, 1H), 6.60 (br s, 2H), 6.55 (d, *J* = 3.3 Hz, 1H), 5.66 (s, 2H), 3.51 (t, *J* = 8.2 Hz, 2H), 0.87 (t, *J* = 8.2 Hz, 2 H), −0.11 (s, 9H) ^13^C NMR (50 MHz, CDCl_3_) δ 169.4, 149.5, 142.9, 129.6, 129.1, 122.4, 120.1, 102.3, 73.2, 66.5, 17.8, -1.4 TLC-MS (ESI) *m*/*z*: 314.4 [M + Na]^+^ HPLC t_ret_ = 7.58 min.

*3-bromo-1-((2-(trimethylsilyl)ethoxy)methyl)-1H-pyrrolo[2,3-b]pyridine-5-carboxamide* (**28**): An ice-cooled solution of 150 mg **27** (0.52 mmol) in 10 mL dry DCM was treated dropwise with a 1 M bromine solution in DCM until a yellow coloration persisted. TLC indicated complete conversion at this point and the reaction was quenched by addition of aqueous Na_2_SO_3_ solution. After dilution with 40 mL EtOAc, the organic phase was washed with sat. NaHCO_3_ and brine, prior to drying over Na2SO4 and evaporation. The residue was purified via flash chromatography (petrol ether/EtOAc + 5% MeOH (20–100%)) to obtain 130 mg (68%) of the title compound as a white solid. ^1^H NMR (200 MHz, CDCl_3_) δ 8.85 (s, 1H), 8.31 (s, 1H), 7.42 (s, 1H), 6.81–6.45 (m, 2H), 5.64 (s, 2H), 3.52 (t, *J* = 8.2 Hz, 2H), 0.88 (t, *J* = 8.2 Hz, 2H), −0.09 (s, 9H) ^13^C NMR (50 MHz, CDCl_3_) δ 168.9, 148.4, 144.2, 128.5, 127.8, 123.1, 119.5, 91.3, 73.2, 66.8, 17.8, −1.4 TLC-MS (ESI) *m*/*z*: 424.3 [M + Na + MeOH]^+^ HPLC t_ret_ = 8.77 min.

*3-phenyl-1-((2-(trimethylsilyl)ethoxy)methyl)-1H-pyrrolo[2,3-b]pyridine-5-carboxamide* (**29**): The preparation was performed following *General Procedure A* from 62 mg **28** (0.17 mmol) and 24 mg phenylboronic acid (0.20 mmol), catalyzed by 2 mg *t*Bu_3_P Pd G3 (3 µmol) in 4 mL dioxane and 1 mL of an 0.5 M K_3_PO_4_ solution. The reaction was conducted at ambient temperature and the crude product was of sufficient purity to be used directly in the next step. Yield: 62 mg (quant.) as a colorless semisolid. ^1^H NMR (200 MHz, CDCl_3_/MeOD) δ 8.85 (d, *J* = 1.6 Hz, 1H), 8.73 (d, *J* = 1.9 Hz, 1H), 7.67–7.56 (m, 3H), 7.50–7.27 (m, 3 H), 5.71 (s, 2 H), 3.59 (t, *J* = 8.3 Hz, 2H), 0.93 (t, *J* = 8.3 Hz, 2H), −0.06 (s, 9H). ^13^C NMR (50 MHz, CDCl_3_/MeOD) δ 169.3, 149.6, 143.0, 133.5, 128.9, 128.6, 127.1, 126.8, 126.2, 122.5, 118.3, 117.7, 73.1, 66.5, 17.6, −1.6. TLC-MS (ESI) *m*/*z*: 422.2 [M + Na + MeOH]^+^ HPLC t_ret_ = 9.44 min.

*3-(3-acrylamidophenyl)-1-((2-(trimethylsilyl)ethoxy)methyl)-1H-pyrrolo[2,3-b]pyridine-5-carboxamide* (**30**): The preparation was performed following *General Procedure A* from 61 mg **28** (0.17 mmol) and 56 mg **14** (0.21 mmol), catalyzed by 3 mg *t*Bu_3_P Pd G3 (5 µmol) in 4 mL dioxane and 1 mL of an 0.5 M K_2_CO_3_ solution. The reaction was conducted at ambient temperature and the crude product was purified via flash chromatography (petrol ether/EtOAc + 5% MeOH (40–100%)). Yield: 67 mg (93%) as colorless a semisolid. ^1^H NMR (200 MHz, DMSO) δ 10.28 (br s, 1H), 8.87 (s, 1H), 8.78 (s, 1H), 8.18 (br s, 1H), 8.07 (s, 1H), 8.03 (s, 1H), 7.77–7.60 (m, 1 H), 7.45 (d, *J* = 4.7 Hz, 3H), 6.49 (dd, *J* = 17.0, 9.8 Hz, 1 H), 6.29 (dd, *J* = 17.0, 1.7 Hz, 1H), 5.85–5.53 (m, 3H), 3.58 (t, *J* = 7.8 Hz, 2H), 0.85 (t, *J* = 7.8 Hz, 2H), −0.10 (s, 9H) ^13^C NMR (50 MHz, DMSO) δ 167.4, 163.3, 149.2, 143.5, 143.5, 139.7, 134.2, 131.9, 129.5, 127.9, 127.8, 127.0, 123.5, 122.0, 117.6, 117.0, 115.6, 72.7, 65.7, 17.2, −1.4 TLC-MS (ESI) *m*/*z*: 459.5 [M + Na]^+^ HPLC t_ret_ = 8.53 min.

*3-phenyl-1H-pyrrolo[2,3-b]pyridine-5-carboxamide* (**10c**): The preparation was carried out following *General Procedure B* starting from 62 mg **29** (0.17 mmol) in 6 mL DCM and 2 mL TFA. Flash purification (DCM/MeOH (8–16%)) afforded 37 mg (92%) of the final compound as a white solid. ^1^H NMR (200 MHz, DMSO) δ 12.18 (br s, 1H), 8.82 (d, *J* = 1.4 Hz, 1H), 8.77 (d, *J* = 1.4 Hz, 1H), 8.18 (br s, 1H), 7.96 (d, *J* = 2.0 Hz, 1H), 7.79 (s, 1H), 7.76 (s, 1H), 7.54–7.21 (m, 4H). ^13^C NMR (50 MHz, DMSO) δ 167.7, 150.2, 143.4, 134.5, 128.9, 127.2, 126.5, 126.0, 125.0, 122.4, 116.4, 115.4. TLC-MS (ESI) *m*/*z*: 238.0 [M + H]^+^ HPLC t_ret_ = 5.19 min.

*3-(3-acrylamidophenyl)-1H-pyrrolo[2,3-b]pyridine-5-carboxamide* (**10a**): The preparation was carried out following *General Procedure B* starting from 90 mg **30** (0.21 mmol) in 8 mL DCM and 2 mL TFA. Flash purification (DCM/MeOH (6–16%)) afforded 45 mg (71%) of the final compound as a white solid. ^1^H NMR (400 MHz, DMSO) δ 12.17 (s, 1H), 10.26 (s, 1H), 8.81 (s, 1H), 8.76 (s, 1H), 8.12 (br s, 1H), 7.99 (s, 1H), 7.88 (s, 1H), 7.68 (d, *J* = 7.4 Hz, 1H), 7.50–7.31 (m, 3H), 6.55–6.42 (m, 1H), 6.35–6.22 (m, 1H), 5.83–5.72 (m, 1H) ^13^C NMR (100 MHz, DMSO) δ 167.7, 163.2, 150.1, 143.2, 139.5, 134.9, 131.9, 129.3, 127.2, 126.7, 124.8, 122.6, 121.9, 117.5, 117.2, 116.4, 115.3 TLC-MS (ESI) *m*/*z*: 329.3 [M + Na]^+^ HPLC t_ret_ = 3.94 min.

*3-bromo-N-cyclopentyl-1-((2-(trimethylsilyl)ethoxy)methyl)-1H-pyrrolo[2,3-b]pyridine-5-carboxamide* (**31a**): The amide coupling was performed following *General Procedure C* starting from 111 mg **18** (0.30 mmol) and 58 mg CDI (0.36 mmol) followed by reaction with 59 µL cyclopentylamine (0.60 mmol). The crude product was purified via flash chromatography (hexane/EtOAc (10–40%)). Yield: 105 mg (80%) as a colorless oil. ^1^H NMR (200 MHz, CDCl_3_) δ 8.73 (d, *J* = 1.9 Hz, 1H), 8.15 (d, *J* = 1.9 Hz, 1H), 7.36 (s, 1H), 6.60 (d, *J* = 7.2 Hz, 1H), 5.58 (s, 2H), 4.49–4.28 (m, 1H), 3.56–3.37 (m, 2H), 2.15–1.93 (m, 2H), 1.80–1.38 (m, 6H), 0.90–0.76 (m, 2H), −0.11 (s, 9H). ^13^C NMR (50 MHz, CDCl_3_) δ 166.2, 148.1, 144.0, 128.1, 126.7, 124.5, 119.1, 90.9, 73.0, 66.6, 51.9, 33.2, 23.9, 23.9, 17.8, −1.4. TLC-MS (ESI) *m*/*z*: 492.3 [M + Na + MeOH]^+^ HPLC t_ret_ = 10.49 min.

*3-bromo-N-methyl-1-((2-(trimethylsilyl)ethoxy)methyl)-1H-pyrrolo[2,3-b]pyridine-5-carboxamide* (**31c**): The amide coupling was performed following *General Procedure C* starting from 100 mg **18** (0.27 mmol) and 53 mg CDI (0.32 mmol) followed by addition with 270 µL of a 2 M solution of methylamine in THF (0.54 mmol). The crude product was purified via flash chromatography (hexane/EtOAc (10–50%)). Yield: 60 mg (58%) as a white solid. ^1^H NMR (200 MHz, CDCl_3_) δ 8.77 (d, *J* = 1.9 Hz, 1H), 8.21 (d, *J* = 2.0 Hz, 1H), 7.38 (s, 1H), 6.90 (d, *J* = 5.1 Hz, 1H), 5.60 (s, 2H), 3.49 (t, *J* = 8.3 Hz, 2H), 3.01 (d, *J* = 4.7 Hz, 3H), 0.86 (t, *J* = 8.2 Hz, 2H), -0.11 (s, 9H). ^13^C NMR (50 MHz, CDCl_3_) δ 167.4, 148.2, 143.9, 128.3, 126.9, 124.2, 119.2, 91.0, 73.1, 66.7, 27.1, 17.8, −1.4 TLC-MS (ESI) *m*/*z*: 405.9 [M + Na]^+^ HPLC t_ret_ = 9.47 min.

*3-bromo-N-ethyl-1-((2-(trimethylsilyl)ethoxy)methyl)-1H-pyrrolo[2,3-b]pyridine-5-carboxamide* (**31d**): The amide coupling was performed following *General Procedure C* starting from 150 mg **18** (0.41 mmol) and 86 mg CDI (0.53 mmol) followed by addition with 1 mL of a 2 M solution of ethylamine in MeOH (2.0 mmol). The crude product was purified via flash chromatography (hexane/EtOAc (10–50%)). Yield: 74 mg (46%) as a yellowish solid. ^1^H NMR (200 MHz, CDCl_3_) δ 9.02 (d, *J* = 1.8 Hz, 1H), 8.53 (d, *J* = 2.0 Hz, 1H), 7.45 (s, 1H), 7.38 (s, 1H), 5.67 (s, 2H), 3.70 (dd, *J* = 16.2, 10.0 Hz, 2H), 3.53 (t, *J* = 8.3 Hz, 2H), 1.01–0.77 (m, 5H), −0.07 (s, 9H). ^13^C NMR (50 MHz, CDCl_3_) δ 166.3, 148.0, 143.6, 128.2, 126.8, 124.2, 119.2, 90.9, 73.0, 66.6, 35.0, 17.7, 14.9, −1.5. HPLC t_ret_ = 9.85 min.

*3-bromo-N-(cyclopropylmethyl)-1-((2-(trimethylsilyl)ethoxy)methyl)-1H-pyrrolo[2,3-b]pyridine-5-carboxamide* (**31e**): The amide coupling was performed following *General Procedure C* starting from 100 mg **18** (0.27 mmol) and 53 mg CDI (0.32 mmol) followed by reaction with 94 µL cyclopropylmethanamine (1.35 mmol). The crude product was purified via flash chromatography (hexane/EtOAc (20–80%)). Yield: 83 mg (80%) as a white solid. ^1^H NMR (200 MHz, CDCl_3_ + MeOD) δ 8.77 (d, *J* = 2.0 Hz, 1H), 8.20 (d, *J* = 2.0 Hz, 1H), 7.44 (s, 1H), 6.42 (s, 1H), 5.65 (s, 2H), 3.70 (s, 2H), 3.60–3.42 (m, 2H), 3.04–2.85 (m, 1H), 0.99–0.80 (m, 4H), 0.76–0.58 (t, 2H), −0.07 (s, 9H). ^13^C NMR (50 MHz, CDCl_3_ + MeOD) δ 168.0, 148.5, 144.0, 128.4, 126.9, 124.1, 119.3, 91.2, 73.1, 67.2, 66.8, 23.4, 17.9, 7.0, −1.3. TLC-MS (ESI) *m*/*z*: 446.0 [M + Na]^+^ HPLC t_ret_ = 9.88 min.

*3-bromo-N-(cyclohexylmethyl)-1-((2-(trimethylsilyl)ethoxy)methyl)-1H-pyrrolo[2,3-b]pyridine-5-carboxamide* (**31f**): The amide coupling was performed following *General Procedure C* starting from 100 mg **18** (0.27 mmol) and 53 mg CDI (0.32 mmol) followed by reaction with 175 µL cyclohexylmethanamine (1.35 mmol). The crude product was purified via flash chromatography (hexane/EtOAc (0–40%)). Yield: 72 mg (53%) as a brown oil. ^1^H NMR (200 MHz, CDCl_3_) δ 8.82 (d, *J* = 2.1 Hz, 1H), 8.26 (d, *J* = 2.1 Hz, 1H), 7.46 (s, 1H), 6.27 (s, 1H), 5.67 (s, 2H), 3.53 (t, 2H), 3.37 (dd, *J* = 7.1, 5.4 Hz, 2H), 1.37 –1.06 (m, 4H), 0.99–0.82 (m, 4H), 0.65–0.53 (m, 2H), 0.33 (t, *J* = 5.2 Hz, 2H), 0.07 (s, 1H), −0.06 (s, 9H). TLC-MS (ESI) *m*/*z*: 488.0 [M + Na]^+^ HPLC t_ret_ = 12.44 min.

*N-benzyl-3-bromo-1-((2-(trimethylsilyl)ethoxy)methyl)-1H-pyrrolo[2,3-b]pyridine-5-carboxamide* (**31g**): The amide coupling was performed following *General Procedure C* starting from 300 mg **18** (0.81 mmol) and 171 mg CDI (1.05 mmol) followed by reaction with 441 µL benzylamine (1.35 mmol). The crude product was purified via flash chromatography (hexane/EtOAc (10–50%)). Yield: 205 mg (55%) as a brown viscous oil. ^1^H NMR (200 MHz, CDCl_3_) δ 8.83 (d, *J* = 2.1 Hz, 1H), 8.26 (d, *J* = 2.0 Hz, 1H), 7.42 (s, 1H), 7.41–7.27 (m, 5H), 6.73 (t, *J* = 5.7 Hz, 1H), 5.64 (s, 2H), 4.67 (d, *J* = 5.6 Hz, 2H), 3.58–3.41 (m, 2H), 0.96–0.81 (m, 2H), −0.07 (s, 9H). TLC-MS (ESI) *m*/*z*: 481.9 [M + Na]^+^ HPLC t_ret_ = 10.60 min.

*3-(3-acrylamidophenyl)-N-cyclopentyl-1-((2-(trimethylsilyl)ethoxy)methyl)-1H-pyrrolo[2,3-b]pyridine-5-carboxamide* (**32a**): The preparation was performed following General Procedure A from 105 mg **31a** (0.24 mmol) and 71 mg **14** (0.26 mmol), catalyzed by 3 mg tBu_3_P Pd G3 (5 µmol) in 5.6 mL dioxane and 1.4 mL of a 0.5 M K_3_PO_4_ solution. The reaction was conducted at ambient temperature and the crude product was purified via flash chromatography (hexane/EtOAc (30–70%)). Yield: 92 mg (78%) as a white solid. ^1^H NMR (200 MHz, DMSO) δ 10.28 (s, 1H), 8.82 (d, *J* = 1.9 Hz, 1H), 8.72 (d, *J* = 1.9 Hz, 1H), 8.44 (d, *J* = 7.3 Hz, 1H), 8.10–8.00 (m, 2H), 7.71–7.60 (m, 1H), 7.52–7.40 (m, 2H), 6.49 (dd, *J* = 17.0, 9.8 Hz, 1H), 6.29 (dd, *J* = 17.0, 2.2 Hz, 1H), 5.79 (dd, *J* = 9.8, 2.2 Hz, 1H), 5.71 (s, 2H), 4.38–4.18 (m, 1H), 3.58 (t, *J* = 7.9 Hz, 2H), 2.02–1.84 (m, 2H), 1.76–1.39 (m, 6H), 0.84 (t, *J* = 7.9 Hz, 2H), −0.10 (s, 9H). ^13^C NMR (50 MHz, DMSO) δ 165.4, 163.3, 149.0, 143.2, 139.7, 134.3, 131.9, 129.5, 127.8, 127.4, 126.9, 124.2, 121.9, 117.6, 117.5, 116.9, 115.6, 72.7, 65.7, 51.0, 32.2, 23.7, 17.2, −1.4. TLC-MS (ESI) *m*/*z*: 527.5 [M + Na]^+^ HPLC t_ret_ = 9.90 min.

*3-(3-acrylamidophenyl)-N-methyl-1-((2-(trimethylsilyl)ethoxy)methyl)-1H-pyrrolo[2,3-b]pyridine-5-carboxamide* (**32c**): The preparation was performed following General Procedure A from 60 mg **31c** (0.16 mmol) and 64 mg **14** (0.23 mmol), catalyzed by 3 mg tBu_3_P Pd G3 (5 µmol) in 4 mL dioxane and 0.9 mL of a 0.5 M K_3_PO_4_ solution. The reaction was conducted at ambient temperature and the crude product was purified via flash chromatography (petrol ether/EtOAc + 5% MeOH (40–100%)). Yield: 48 mg (69%) as a white solid. The material was carried on directly to the next step. TLC-MS (ESI) *m*/*z*: 473.0 [M + Na]^+^ HPLC t_ret_ = 8.97 min.

*3-(3-acrylamidophenyl)-N-ethyl-1-((2-(trimethylsilyl)ethoxy)methyl)-1H-pyrrolo[2,3-b]pyridine-5-carboxamide* (**32d**): The preparation was performed following General Procedure A from 74 mg **31d** (0.19 mmol) and 64 mg **14** (0.23 mmol), catalyzed by 3 mg tBu_3_P Pd G3 (5 µmol) in 4 mL dioxane and 1.1 mL of a 0.5 M K_2_CO_3_ solution. The reaction was conducted at ambient temperature and the crude product was purified via flash chromatography (petrol ether/EtOAc (40–100%)). Yield: 48 mg (69%) as white solid. The material was carried on directly to the next step. TLC-MS (ESI) *m*/*z*: 487.2 [M + Na]^+^ HPLC t_ret_ = 9.11 min.

*3-(3-acrylamidophenyl)-N-(cyclopropylmethyl)-1-((2-(trimethylsilyl)ethoxy)methyl)-1H-pyrrolo[2,3-b]pyridine-5-carboxamide* (**32e**): The preparation was performed following General Procedure A from 83 mg **31e** (0.20 mmol) and 83 mg **14** (0.30 mmol), catalyzed by 3 mg tBu_3_P Pd G3 (5 µmol) in 5 mL dioxane and 1.2 mL of a 0.5 M K_2_CO_3_ solution. The reaction was conducted at ambient temperature and the crude product was purified via flash chromatography (petrol ether/EtOAc + 5% MeOH (40–100%)). Yield: 78 mg (59%) as a white solid. ^1^H NMR (200 MHz, CDCl_3_) δ 8.82 (d, *J* = 2.0 Hz, 1H), 8.68 (d, *J* = 2.0 Hz, 1H), 8.08 (s, 1H), 7.93 (s, 1H), 7.55 (s, 1H), 7.49 (s, 1H), 7.34 (q, *J* = 7.4 Hz, 2H), 6.73 (t, *J* = 5.5 Hz, 1H), 6.55–6.33 (m, 2H), 5.76 (dd, *J* = 8.3, 3.3 Hz, 1H), 5.69 (s, 2H), 3.56 (t, 2H), 3.37 (t, 2H), 1.13 (d, *J* = 9.8 Hz, 1H), 1.01–0.82 (m, 2H), 0.65–0.46 (m, 2H), 0.30 (d, *J* = 5.1 Hz, 2H), −0.07 (s, 9H). TLC-MS (ESI) *m*/*z*: 513.2 [M + Na]^+^ HPLC t_ret_ = 8.82 min.

*3-(3-acrylamidophenyl)-N-(cyclohexylmethyl)-1-((2-(trimethylsilyl)ethoxy)methyl)-1H-pyrrolo[2,3-b]pyridine-5-carboxamide* (**32f**): The preparation was performed following General Procedure A from 72 mg **31f** (0.16 mmol) and 64 mg **14** (0.23 mmol), catalyzed by 3 mg tBu_3_P Pd G3 (5 µmol) in 4 mL dioxane and 0.9 mL of a 0.5 M K_2_CO_3_ solution. The reaction was conducted at ambient temperature and the crude product was purified via flash chromatography (petrol ether/EtOAc (20–60%)). Yield: 57 mg (69%) as a yellowish solid. ^1^H NMR (200 MHz, CDCl_3_) δ 8.81 (s, 1H), 8.70 (s, 1H), 8.10 (s, 1H), 7.99 (s, 1H), 7.78–7.30 (m, 4H), 6.74 (s, 1H), 6.42 (s, 1H), 6.37 (s, 1H), 5.77 (s, 1H), 5.68 (s, 2H), 3.55 (t, 2H), 3.34 (t, 2H), 1.73 (d, *J* = 16.2 Hz, 9H), 1.10 (s, 1H), 0.91 (t, *J* = 8.1 Hz, 2H), 0.07 (s, 1H), −0.07 (s, 9H). TLC-MS (ESI) *m*/*z*: 555.3 [M + Na]^+^ HPLC t_ret_ = 10.98 min.

*3-(3-acrylamidophenyl)-N-benzyl-1-((2-(trimethylsilyl)ethoxy)methyl)-1H-pyrrolo[2,3-b]pyridine-5-carboxamide* (**32g**): The preparation was performed following General Procedure A from 200 mg **31g** (0.43 mmol) and 149 mg **14** (0.54 mmol), catalyzed by 8 mg tBu_3_P Pd G3 (13 µmol) in 12 mL dioxane and 2.6 mL of a 0.5 M K_2_CO_3_ solution. The reaction was conducted at ambient temperature and the crude product was purified via flash chromatography (petrol ether/EtOAc + 5% MeOH (40–100%)). Yield: 120 mg (53%) as a brown solid. ^1^H NMR (200 MHz, CDCl_3_ + MeOD) δ 8.61 (s, 1H), 8.51 (s, 1H), 8.36 (t, *J* = 5.4 Hz, 1H), 7.78 (s, 1H), 7.36 (s, 1H), 7.30–7.19 (m, 1H), 7.17–6.95 (m, 8H), 6.18–6.10 (m, 2H), 5.51–5.44 (m, 1H), 5.41 (s, 2H), 4.08 (s, 2H), 3.33 (t, *J* = 8.2 Hz, 2H), 0.66 (t, *J* = 8.2 Hz, 2H), −0.31 (s, 9H). ^13^C NMR (50 MHz, CDCl3 + MeOD) δ 167.4, 164.7, 149.2, 143.1, 138.6, 138.3, 134.1, 130.9, 129.3, 128.3, 127.9, 127.4, 127.3, 127.0, 126.4, 123.3, 122.8, 118.7, 118.3, 118.1, 117.0, 73.0, 66.4, 43.6, 17.5, −1.9. TLC-MS (ESI) *m*/*z*: 548.9 [M+Na]^+^ HPLC t_ret_ = 10.08 min.

*3-(3-acrylamidophenyl)-N-cyclopentyl-1H-pyrrolo[2,3-b]pyridine-5-carboxamide* (**11a**): The preparation was carried out following *General Procedure B* starting from 81 mg **32a** (0.16 mmol) in 7.5 mL DCM and 2.5 mL TFA. Flash purification (DCM/MeOH (4–12%)) afforded 49 mg (82%) of the final compound as a white solid. ^1^H NMR (400 MHz, DMSO) δ 12.16 (br s, 1H), 10.26 (s, 1H), 8.77 (d, *J* = 1.6 Hz, 1H), 8.70 (d, *J* = 1.6 Hz, 1H), 8.39 (d, *J* = 7.1 Hz, 1H), 8.06–7.95 (m, 1H), 7.88 (d, *J* = 2.4 Hz, 1H), 7.72–7.61 (m, 1H), 7.49–7.34 (m, 2H), 6.48 (dd, *J* = 17.0, 10.1 Hz, 1H), 6.29 (dd, *J* = 17.0, 1.8 Hz, 1H), 5.78 (dd, *J* = 10.1, 1.8 Hz, 1H), 4.34–4.20 (m, 1H), 2.00–1.81 (m, 2H), 1.79–1.64 (m, 2H), 1.63–1.46 (m, 4H). ^13^C NMR (101 MHz, DMSO) δ 165.6, 163.2, 149.9, 142.9, 139.5, 135.0, 131.9, 129.3, 126.9, 126.7, 124.8, 123.3, 121.8, 117.5, 117.1, 116.3, 115.3, 51.0, 32.1, 23.6. TLC-MS (ESI) *m*/*z*: 397.0 [M + Na]^+^ HPLC t_ret_ = 6.94 min.

*3-(3-acrylamidophenyl)-N-methyl-1H-pyrrolo[2,3-b]pyridine-5-carboxamide* (**11c**): The preparation was carried out following *General Procedure B* starting from 48 mg **32c** (0.11 mmol) in 7 mL DCM and 3 mL TFA. Flash purification (DCM/MeOH (6–16%)) afforded 31 mg (91%) of the final compound as a white solid. ^1^H NMR (400 MHz, DMSO-*d*_6_) δ 12.18 (s, 1H), 10.27 (s, 1H), 8.76 (d, *J* = 2.0 Hz, 1H), 8.71 (d, *J* = 2.0 Hz, 1H), 8.57 (d, *J* = 4.6 Hz, 1H), 7.98 (s, 1H), 7.89 (d, *J* = 2.6 Hz, 1H), 7.69 (d, *J* = 7.5 Hz, 1H), 7.51–7.37 (m, 2H), 6.49 (dd, *J* = 16.9, 10.1 Hz, 1H), 6.29 (dd, *J* = 16.9, 2.0 Hz, 1H), 5.78 (dd, 1H), 2.83 (d, *J* = 4.4 Hz, 3H) ^13^C NMR (101 MHz, DMSO-*d*_6_) δ 166.9, 163.7, 150.5., 143.3, 140.0, 135.4, 132.5, 129.8, 127.3, 127.1, 125.4, 123.5, 122.4, 118.0, 117.7, 116.9, 115.8, 26.7. TLC-MS (ESI) *m*/*z*: 343.0 [M + Na]^+^. HRMS ESI-TOF [M + H]^+^ *m*/*z* calcd. for C_18_H_16_N_4_O_2_: 321.1346, found: 321.1351. HPLC t_ret_ = 4.86 min.

*3-(3-acrylamidophenyl)-N-ethyl-1H-pyrrolo[2,3-b]pyridine-5-carboxamide* (**11d**): The preparation was carried out following *General Procedure B* starting from 68 mg **32d** (0.15 mmol) in 4.8 mL DCM and 1.2 mL TFA. Flash purification (DCM/MeOH (6–10%)) afforded 34 mg (70%) of the final compound as a white solid. ^1^H NMR (400 MHz, DMSO-*d*_6_) δ 12.16 (s, 1H), 10.25 (s, 1H), 8.77 (d, *J* = 2.0 Hz, 1H), 8.71 (d, *J* = 2.0 Hz, 1H), 8.58 (t, *J* = 5.5 Hz, 1H), 7.98 (d, *J* = 1.7 Hz, 1H), 7.88 (d, *J* = 2.6 Hz, 1H), 7.68 (dt, *J* = 7.1, 2.1 Hz, 1H), 7.49–7.38 (m, 2H), 6.48 (dd, *J* = 16.9, 10.1 Hz, 1H), 6.29 (dd, *J* = 17.0, 2.1 Hz, 1H), 5.78 (dd, *J* = 10.1, 2.1 Hz, 1H), 3.40–3.33 (m, 2H), 1.16 (t, *J* = 7.2 Hz, 3H). ^13^C NMR (101 MHz, DMSO-*d*_6_) δ 166.2, 163.7, 150.5, 143.3, 140.0, 135.5, 132.4, 129.8, 127.3, 127.2, 125.4, 123.6, 122.4, 118.0, 117.7, 116.9, 115.8, 34.5, 15.3. TLC-MS (ESI) *m*/*z*: 357.0 [M + Na]^+^. HRMS ESI-TOF [M + H]^+^ *m*/*z* calcd. for C_19_H_18_N_4_O_2_: 335,1503, found: 335.1508. HPLC t_ret_ = 5.53 min.

*3-(3-acrylamidophenyl)-N-(cyclopropylmethyl)-1H-pyrrolo[2,3-b]pyridine-5-carboxamide* (**11e**): The preparation was carried out following *General Procedure B* starting from 78 mg **32e** (0.16 mmol) in 4.2 mL DCM and 1.8 mL TFA. Flash purification (DCM/MeOH (6–16%)) afforded 13 mg (23%) of the final compound as a yellowish solid. ^1^H NMR (400 MHz, DMSO-*d*_6_) δ 12.18 (s, 1H), 10.30 (s, 1H), 8.80 (d, *J* = 2.0 Hz, 1H), 8.74 (d, *J* = 2.1 Hz, 1H), 8.71 (t, *J* = 5.6 Hz, 1H), 8.00 (d, *J* = 2.2 Hz, 1H), 7.90 (s, 1H), 7.70 (dt, *J* = 7.3, 1.9 Hz, 1H), 7.50–7.38 (m, 2H), 6.49 (dd, *J* = 17.0, 10.1 Hz, 1H), 6.29 (dd, *J* = 17.0, 2.1 Hz, 1H), 5.79 (dd, *J* = 10.0, 2.1 Hz, 1H), 3.20 (t, *J* = 6.2 Hz, 2H), 1.07 (ttd, *J* = 12.9, 6.8, 3.7 Hz, 1H), 0.51–0.37 (m, 2H), 0.36–0.22 (m, 2H). ^13^C NMR (101 MHz, DMSO-*d*_6_) δ 165.8, 163.2, 149.9, 142.8, 139.5, 134.9, 131.9, 129.2, 126.8, 126.7, 124.8, 123.0, 121.9, 117.5, 117.1, 116.4, 115.3, 43.5, 11.0, 3.3. TLC-MS (ESI) *m*/*z*: 383.1 [M + Na]^+^. HRMS ESI-TOF [M + H]^+^ *m*/*z* calcd. for C_21_H_20_N_4_O_2_: 361.1659, found: 361.1662. HPLC t_ret_ = 6.54 min.

*3-(3-acrylamidophenyl)-N-(cyclohexylmethyl)-1H-pyrrolo[2,3-b]pyridine-5-carboxamide* (**11f**): The preparation was carried out following *General Procedure B* starting from 30 mg **32f** (0.06 mmol) in 7 mL DCM and 3 mL TFA. Flash purification (DCM/MeOH (5–10%)) afforded 15 mg (64%) of the final compound as a yellowish solid. ^1^H NMR (400 MHz, DMSO-*d*_6_) δ 12.16 (s, 1H), 10.27 (s, 1H), 8.78 (s, 1H), 8.72 (s, 1H), 8.54 (s, 1H), 8.02 (s, 1H), 7.88 (d, *J* = 3.0 Hz, 1H), 7.66 (d, *J* = 7.2 Hz, 1H), 7.44 (s, 2H), 6.49 (ddd, *J* = 17.2, 10.1, 3.3 Hz, 1H), 6.29 (d, *J* = 16.8 Hz, 1H), 5.78 (d, *J* = 10.2 Hz, 1H), 3.16 (q, *J* = 6.5, 5.0 Hz, 2H), 1.72 (dd, *J* = 24.7, 11.6 Hz, 4H), 1.61 (d, *J* = 10.0 Hz, 2H), 1.22 (s, 1H), 1.17 (d, *J* = 9.2 Hz, 2H), 0.95 (t, *J* = 12.0 Hz, 2H). ^13^C NMR (101 MHz, DMSO-*d*_6_) δ 165.9, 163.2, 149.9, 142.8, 139.5, 134.9, 131.9, 129.2, 126.7, 126.6, 124.8, 123.1, 121.8, 117.5, 117.1, 116.3, 115.2, 45.4, 37.4, 30.5, 26.0, 25.4. TLC-MS (ESI) *m*/*z*: 425.3 [M + Na]^+^. HRMS ESI-TOF [M + H]^+^ *m*/*z* calcd. for C_24_H_26_N_4_O_2_: 403.2129, found: 403.2133. HPLC t_ret_ = 8.50 min.

*3-(3-acrylamidophenyl)-N-benzyl-1H-pyrrolo[2,3-b]pyridine-5-carboxamide* (**11g**): The preparation was carried out following *General Procedure B* starting from 80 mg **32g** (0.15 mmol) in 7 mL DCM and 3 mL TFA. Flash purification (DCM/MeOH (6–8%)) afforded 58 mg (97%) of the final compound as a pale red solid. ^1^H NMR (400 MHz, DMSO-*d*_6_) δ 12.20 (d, *J* = 2.7 Hz, 1H), 10.26 (s, 1H), 9.18 (t, *J* = 6.0 Hz, 1H), 8.84 (d, *J* = 2.0 Hz, 1H), 8.79 (d, *J* = 2.0 Hz, 1H), 8.00 (t, *J* = 1.9 Hz, 1H), 7.90 (d, *J* = 2.6 Hz, 1H), 7.67 (dt, *J* = 7.7, 1.8 Hz, 1H), 7.52–7.18 (m, 7H), 6.48 (q, *J* = 16.9, 10.1 Hz, 1H), 6.29 (dd, *J* = 17.0, 2.1 Hz, 1H), 5.78 (dd, *J* = 10.1, 2.0 Hz, 1H), 4.54 (d, *J* = 5.9 Hz, 2H). ^13^C NMR (101 MHz, DMSO-*d*_6_) δ 166.0, 163.2, 150.1, 142.9, 139.7, 139.5, 134.9, 131.9, 129.3, 128.2, 127.2, 126.9, 126.8, 126.7, 124.9, 122.7, 121.9, 117.5, 117.2, 116.4, 115.4, 42.6. TLC-MS (ESI) *m*/*z*: 419.3 [M + Na]^+^. HRMS ESI-TOF [M + H]^+^ *m*/*z* calcd. for C_24_H_20_N_4_O_2_: 397.1659, found: 397.1663. HPLC t_ret_ = 7.20 min.

*N-(3-bromo-1-((2-(trimethylsilyl)ethoxy)methyl)-1H-pyrrolo[2,3-b]pyridin-5-yl)cyclopentanecarboxamide* (**33a**): The preparation was carried out following *General Procedure D* with 90 mg **22** (0.26 mmol), 111 µL Et_3_N (0.79 mmol) and 70 mg cyclopentanecarboxylic acid chloride (0.53 mmol). Flash purification (hexane/EtOAc (10–50%)) afforded 90 mg (78%) of the product as a colorless oily residue. ^1^H NMR (200 MHz, CDCl_3_) δ 8.30–8.21 (m, 1H), 8.20–8.15 (m, 1H), 8.11 (br s, 1H), 7.29 (s, 1H), 5.54 (s, 2H), 3.48 (t, *J* = 8.2 Hz, 2H), 2.82–2.60 (m, 1H), 1.95–1.80 (m, 4H), 1.78–1.43 (m, 4H), 0.86 (t, *J* = 8.2 Hz, 2H), −0.10 (s, 9H). ^13^C NMR (50 MHz, CDCl_3_) δ 175.6, 144.1, 138.3, 129.5, 127.6, 120.0, 119.7, 89.9, 73.0, 66.5, 46.4, 30.7, 26.1, 17.8, −1.4. TLC-MS (ESI) *m*/*z*: 492.1 [M + Na + MeOH]^+^ HPLC t_ret_ = 10.84 min.

*N-(3-bromo-1-((2-(trimethylsilyl)ethoxy)methyl)-1H-pyrrolo[2,3-b]pyridin-5-yl)thiophene-2-carboxamide* (**33c**): The preparation was carried out following *General Procedure D* with 100 mg **22** (0.29 mmol), 123 µL Et_3_N (0.88 mmol) and 41 µL thiophene-2-carbonyl chloride (0.38 mmol). Flash purification (petrol ether/EtOAc + 10% THF (0–50%)) afforded 62 mg (47%) of the product as a white solid. ^1^H NMR (200 MHz, CDCl_3_) δ 8.39 (d, *J* = 2.3 Hz, 1H), 8.24 (d, *J* = 2.3 Hz, 1H), 8.22 (s, 1H), 7.71 (dd, *J* = 3.8, 1.1 Hz, 1H), 7.54 (dd, *J* = 5.0, 1.1 Hz, 1H), 7.36 (s, 1H), 7.10 (dd, *J* = 5.0, 3.7 Hz, 1H), 5.59 (s, 2H), 3.52 (t, 2H), 0.89 (t, *J* = 11.1, 2.2, 0.9 Hz, 2H), −0.07 (s, 9H). ^13^C NMR (50 MHz, CDCl_3_) δ 161.4, 145.1, 139.5, 139.3, 131.8, 129.6, 129.5, 128.6, 121.5, 120.6, 90.8, 73.8, 67.3, 18.5, −0.7. TLC-MS (ESI) *m*/*z*: 474.3 [M + Na]^+^ HPLC t_ret_ = 10.07 min.

*N-(3-bromo-1-((2-(trimethylsilyl)ethoxy)methyl)-1H-pyrrolo[2,3-b]pyridin-5-yl)furan-2-carboxamide* (**33d**): The preparation was carried out following *General Procedure D* with 100 mg **22** (0.29 mmol), 123 µL Et_3_N (0.88 mmol) and 44 µL furane-2-carbonyl chloride (0.38 mmol). Flash purification (petrol ether/EtOAc (40–50%)) afforded 101 mg (80%) of the product as a yellow oil. ^1^H NMR (200 MHz, CDCl_3_) δ 8.44 (d, *J* = 2.4 Hz, 1H), 8.33 (d, *J* = 2.4 Hz, 1H), 8.29 (s, 1H), 7.51 (dd, *J* = 1.7, 0.8 Hz, 1H), 7.37 (s, 1H), 7.25 (dd, *J* = 3.3, 1.1 Hz, 1H), 6.56 (dd, *J* = 3.6, 1.8 Hz, 1H), 5.61 (s, 2H), 3.52 (t, 2H), 0.89 (t, 2H), −0.07 (s, 9H). ^13^C NMR (50 MHz, CDCl_3_) δ 156.4, 147.5, 144.4, 144.3, 138.0, 128.5, 127.8, 119.9, 119.7, 115.4, 112.6, 89.9, 72.9, 66.4, 17.7, −1.5. TLC-MS (ESI) *m*/*z*: 458.1 [M + Na]^+^ HPLC t_ret_ = 9.92 min.

*N-(3-bromo-1-((2-(trimethylsilyl)ethoxy)methyl)-1H-pyrrolo[2,3-b]pyridin-5-yl)benzamide* (**33e**): The preparation was carried out following *General Procedure D* with 100 mg **22** (0.29 mmol), 123 µL Et_3_N (0.88 mmol) and 68 µL benzoyl chloride (0.58 mmol). Flash purification (petrol ether/EtOAc (30–60%)) afforded 91 mg (70%) of the product as a yellow solid. ^1^H NMR (200 MHz, CDCl_3_) δ 8.40 (s, 1H), 8.38 (s, 1H), 8.28 (s, 1H), 7.90 (d, *J* = 7.5 Hz, 2H), 7.49 (q, *J* = 13.4, 11.4 Hz, 3H), 7.35 (s, 1H), 5.59 (s, 2H), 3.52 (t, *J* = 8.3 Hz, 2H), 0.89 (t, *J* = 8.0 Hz, 2H), −0.07 (s, 9H). ^13^C NMR (50 MHz, CDCl_3_) δ 166.2, 144.3, 138.5, 134.3, 131.9, 129.0, 128.7, 127.8, 127.1, 120.5, 119.7, 89.9, 72.9, 66.4, 17.7, −1.5. TLC-MS (ESI) *m*/*z*: 468.2 [M + Na]^+^ HPLC t_ret_ = 10.20 min.

*N-(3-bromo-1-((2-(trimethylsilyl)ethoxy)methyl)-1H-pyrrolo[2,3-b]pyridin-5-yl)acetamide* (**33f**): The preparation was carried out following *General Procedure D* with 130 mg **22** (0.38 mmol), 160 µL Et_3_N (1.14 mmol) and 54 µL acetyl chloride (0.76 mmol). Flash purification (petrol ether/EtOAc (30–80%)) afforded 134 mg (83%) of the product as a yellow solid. ^1^H NMR (200 MHz, CDCl_3_) δ 8.25 (d, *J* = 2.3 Hz, 1H), 8.19 (s, 1H), 8.12 (d, *J* = 2.3 Hz, 1H), 7.32 (s, 1H), 5.56 (s, 2H), 3.59–3.40 (t, 2H), 2.19 (s, 3H), 0.99–0.73 (t, 2H), −0.08 (s, 9H). ^13^C NMR (50 MHz, CDCl_3_) δ 169.4, 144.3, 138.4, 129.2, 127.9, 120.4, 119.8, 90.0, 73.1, 66.6, 24.3, 17.8, −1.4. TLC-MS (ESI) *m*/*z*: 406.1 [M + Na]^+^ HPLC t_ret_ = 9.44 min.

*N-(3-bromo-1-((2-(trimethylsilyl)ethoxy)methyl)-1H-pyrrolo[2,3-b]pyridin-5-yl)-2-cyclopentylacetamide* (**33g**): The preparation was carried out following *General Procedure D* with 100 mg **22** (0.29 mmol), 123 µL Et_3_N (0.88 mmol) and 79 µL 2-cyclopentylacetyl chloride (0.58 mmol). Flash purification (petrol ether/EtOAc (0–40%)) afforded 120 mg (91%) of the product as a brown oil. ^1^H NMR (200 MHz, CDCl_3_) δ 8.30 (d, *J* = 2.2 Hz, 1H), 8.27 (d, *J* = 2.1 Hz, 1H), 7.68–7.52 (m, 1H), 7.36 (s, 1H), 5.59 (s, 2H), 3.65–3.36 (m, 2H), 2.36 (d, *J* = 6.5 Hz, 2H), 2.23 (p, *J* = 7.3 Hz, 1H), 2.01–1.75 (m, 4H), 1.72–1.45 (m, 4H), 0.91 (td, *J* = 8.3, 7.7, 4.8 Hz, 2H), −0.06 (d, *J* = 2.5 Hz, 9H). TLC-MS (ESI) *m*/*z*: 474.2 [M + Na]^+^ HPLC t_ret_ = 11.32 min.

*N-(3-bromo-1-((2-(trimethylsilyl)ethoxy)methyl)-1H-pyrrolo[2,3-b]pyridin-5-yl)-2-phenylacetamide* (**33h**): The preparation was carried out following *General Procedure D* with 100 mg **22** (0.29 mmol), 123 µL Et_3_N (0.88 mmol) and 77 µL 2-phenylacetyl chloride (0.58 mmol). Flash purification (petrol ether/EtOAc (40–70%)) afforded 111 mg (82%) of the product as a yellow oil. ^1^H NMR (200 MHz, CDCl_3_) δ 8.18 (d, *J* = 2.4 Hz, 1H), 8.11 (d, *J* = 2.4 Hz, 1H), 7.45 (d, *J* = 2.5 Hz, 1H), 7.41 (d, *J* = 3.4 Hz, 2H), 7.37 (d, *J* = 2.1 Hz, 2H), 7.34 (s, 2H), 5.57 (s, 2H), 3.77 (s, 2H), 3.49 (t, 2H), 0.88 (t, *J* = 8.2 Hz, 2H), −0.07 (s, 9H). ^13^C NMR (50 MHz, CDCl_3_) δ 169.6, 144.4, 138.2, 134.3, 129.5, 129.2, 128.7, 127.7, 127.6, 120.1, 119.5, 89.8, 72.9, 66.4, 44.4, 17.7, −1.5. TLC-MS (ESI) *m*/*z*: 482.1 [M + Na]^+^ HPLC t_ret_ = 10.42 min.

*N-(3-bromo-1-((2-(trimethylsilyl)ethoxy)methyl)-1H-pyrrolo[2,3-b]pyridin-5-yl)-2-(thiophen-2-yl)acetamide* (**33i**): The preparation was carried out following *General Procedure D* with 100 mg **22** (0.29 mmol), 123 µL Et_3_N (0.88 mmol) and 72 µL 2-(thiophen-2-yl)acetyl chloride (0.58 mmol). Flash purification (petrol ether/EtOAc (20–50%)) afforded 123 mg (90%) of the product as a brown oil. ^1^H NMR (200 MHz, CDCl_3_) δ 8.22 (d, *J* = 2.3 Hz, 1H), 8.12 (d, *J* = 2.3 Hz, 1H), 7.73 (s, 1H), 7.34 (s, 1H), 7.30 (dd, 1H), 7.05 (s, 1H), 7.03 (d, *J* = 1.6 Hz, 1H), 5.57 (s, 2H), 3.97 (s, 2H), 3.49 (t, 2H), 0.88 (t, 2H), −0.08 (s, 9H). ^13^C NMR (50 MHz, CDCl_3_) δ 168.6, 144.5, 138.4, 135.6, 128.7, 128.0, 127.9, 127.7, 126.1, 120.4, 119.8, 90.0, 73.1, 66.6, 38.3, 17.9, −1.3. TLC-MS (ESI) *m*/*z*: 488.1 [M + Na]^+^ HPLC t_ret_ = 10.15 min.

*N-(3-(3-acrylamidophenyl)-1-((2-(trimethylsilyl)ethoxy)methyl)-1H-pyrrolo[2,3-b]pyridin-5-yl)cyclopentanecarboxamide* (**34a**): The preparation was performed following General Procedure A from 90 mg **33a** (0.21 mmol) and 64 mg **14** (0.24 mmol), catalyzed by 2 mg tBu_3_P Pd G3 (4 µmol) in 3.3 mL dioxane and 0.4 mL of a 1.5 M K_2_CO_3_ solution. The reaction was conducted at ambient temperature and the crude product was purified via flash chromatography (hexane/EtOAc (30–80%)). Yield: 76 mg (73%) as a white solid. ^1^H NMR (200 MHz, DMSO) δ 10.25 (s, 1H), 10.04 (s, 1H), 8.57 (d, *J* = 2.1 Hz, 1H), 8.49 (d, *J* = 2.1 Hz, 1H), 8.02–7.95 (m, 1H), 7.93 (s, 1H), 7.66–7.56 (m, 1H), 7.43 (t, *J* = 7.7 Hz, 1H), 7.38–7.28 (m, 1H), 6.48 (dd, *J* = 16.9, 9.9 Hz, 1H), 6.28 (dd, *J* = 16.9, 2.2 Hz, 1H), 5.78 (dd, *J* = 9.9, 2.2 Hz, 1H), 5.65 (s, 2H), 3.56 (t, *J* = 8.0 Hz, 2H), 2.91–2.70 (m, 1H), 1.97–1.46 (m, 8H), 0.84 (t, *J* = 8.0 Hz, 2H), −0.10 (s, 9H). ^13^C NMR (50 MHz, DMSO) δ 174.6, 163.3, 144.8, 139.6, 136.7, 134.8, 131.9, 130.6, 129.4, 127.3, 127.0, 121.8, 121.8, 118.6, 117.4, 117.3, 114.5, 72.6, 65.6, 45.1, 30.1, 25.7, 17.2, −1.4. TLC-MS (ESI) *m*/*z*: 527.1 [M + Na]^+^ HPLC t_ret_ = 10.24 min.

*N-(3-(3-acrylamidophenyl)-1-((2-(trimethylsilyl)ethoxy)methyl)-1H-pyrrolo[2,3-b]pyridin-5-yl)thiophene-2-carboxamide* (**34c**): The preparation was performed following General Procedure A from 90 mg **33c** (0.20 mmol) and 81 mg **14** (0.30 mmol), catalyzed by 3 mg tBu_3_P Pd G3 (5 µmol) in 5.0 mL dioxane and 1.2 mL of a 0.5 M K_2_CO_3_ solution. The reaction was conducted at ambient temperature and the crude product was purified via flash chromatography (petrol ether/EtOAc + 10% THF (20–50%)). Yield: 46 mg (45%) as a white solid. ^1^H NMR (200 MHz, DMSO-*d*_6_) δ 10.44 (s, 1H), 10.24 (s, 1H), 8.61 (s, 1H), 8.60 (s, 1H), 8.14–7.97 (m, 2H), 7.99 (s, 1H), 7.87 (d, *J* = 5.0 Hz, 1H), 7.63 (d, *J* = 7.4 Hz, 1H), 7.42 (d, *J* = 8.1 Hz, 2H), 7.25 (t, *J* = 4.4 Hz, 1H), 6.48 (dd, *J* = 16.9, 9.9 Hz, 1H), 6.27 (dd, *J* = 16.8, 1.7 Hz, 1H), 5.77 (d, *J* = 10.0 Hz, 1H), 5.68 (s, 2H), 3.59 (t, *J* = 7.9 Hz, 2H), 0.86 (t, *J* = 7.9 Hz, 2H), −0.08 (s, 9H). ^13^C NMR (50 MHz, -CDCl_3_ + MeOD) δ 164.8, 161.5, 145.6, 139.2, 138.7, 137.7, 134.7, 131.2, 131.1, 129.5, 129.2, 128.9, 127.9, 127.4, 126.1, 122.4, 121.5, 118.5, 118.3, 118.0, 115.9, 73.2, 66.4, 17.7, −1.7 TLC-MS (ESI) *m*/*z*: 541.5 [M + Na]^+^ HPLC t_ret_ = 9.48 min.

*N-(3-(3-acrylamidophenyl)-1-((2-(trimethylsilyl)ethoxy)methyl)-1H-pyrrolo[2,3-b]pyridin-5-yl)furan-2-carboxamide* (**34d**): The preparation was performed following General Procedure A from 101 mg **33d** (0.23 mmol) and 95 mg **14** (0.35 mmol), catalyzed by 4 mg tBu_3_P Pd G3 (7 µmol) in 6.0 mL dioxane and 1.4 mL of a 0.5 M K_2_CO_3_ solution. The reaction was conducted at ambient temperature and the crude product was purified via flash chromatography (petrol ether/EtOAc + 10% MeOH (40–60%)). Yield: 58 mg (50%) as a white solid. ^1^H NMR (200 MHz, CDCl_3_ + MeOD) δ 9.38 (s, 1H), 9.19 (s, 1H), 8.63 (d, *J* = 2.3 Hz, 1H), 8.46 (d, *J* = 2.3 Hz, 1H), 7.85 (s, 1H), 7.65 (d, *J* = 7.0 Hz, 1H), 7.59–7.46 (m, 2H), 7.40–7.23 (m, 2H), 7.22 (dd, *J* = 3.5, 0.8 Hz, 1H), 6.51 (dd, *J* = 3.5, 1.8 Hz, 1H), 6.38 (s, 1H), 6.35 (d, *J* = 3.3 Hz, 1H), 5.69 (dd, *J* = 7.5, 4.3 Hz, 1H), 5.60 (s, 2H), 3.62–3.43 (m, 2H), 0.96–0.78 (m, 2H), −0.12 (s, 9H). ^13^C NMR (50 MHz, CDCl_3_ + MeOD) δ 222.3, 221.4, 164.6, 147.5, 145.9, 144.9, 138.9, 137.4, 134.8, 131.3, 129.6, 128.4, 127.5, 126.3, 122.4, 121.2, 118.5, 118.2, 116.0, 115.5, 112.5, 73.2, 66.5, 17.8, −1.5. TLC-MS (ESI) *m*/*z*: 525.4 [M + Na]^+^ HPLC t_ret_ = 9.34 min.

*N-(3-(3-acrylamidophenyl)-1-((2-(trimethylsilyl)ethoxy)methyl)-1H-pyrrolo[2,3-b]pyridin-5-yl)benzamide* (**34e**): The preparation was performed following *General Procedure A* from 91 mg **33e** (0.20 mmol) and 84 mg **14** (0.31 mmol), catalyzed by 4 mg *t*Bu_3_P Pd G3 (7 µmol) in 5.0 mL dioxane and 1.2 mL of a 0.5 M K_2_CO_3_ solution. The reaction was conducted at ambient temperature and the crude product was purified via flash chromatography (petrol ether/EtOAc + 10% MeOH (20–60%)). Yield: 47 mg (45%) as a white solid. ^1^H NMR (200 MHz, CDCl_3_ + MeOD) δ 9.65 (s, 1H), 9.47 (s, 1H), 8.57 (d, *J* = 2.1 Hz, 1H), 8.49 (d, *J* = 2.1 Hz, 1H), 7.99–7.81 (m, 3H), 7.63–7.25 (m, 6H), 7.23 (s, 1H), 6.32 (s, 1H), 6.29 (d, *J* = 1.5 Hz, 1H), 5.63 (dd, *J* = 6.6, 5.1 Hz, 1H), 5.55 (s, 2H), 3.47 (t, 2H), 0.81 (t, *J* = 8.3 Hz, 2H), −0.17 (s, 9H). ^13^C NMR (50 MHz, CDCl_3_ + MeOD) δ 224.0, 167.3, 164.7, 145.7, 138.8, 137.7, 134.8, 134.5, 131.9, 131.2, 129.5, 129.4, 128.6, 127.5, 126.1, 122.3, 121.5, 118.5, 118.3, 118.0, 116.0, 73.2, 66.5, 17.8, −1.6. TLC-MS (ESI) *m*/*z*: 535.1 [M + Na]^+^ HPLC t_ret_ = 9.77 min.

*N-(3-(5-acetamido-1-((2-(trimethylsilyl)ethoxy)methyl)-1H-pyrrolo[2,3-b]pyridin-3-yl)phenyl)acrylamide* (**34f**): The preparation was performed following *General Procedure A* from 121 mg **33f** (0.32 mmol) and 129 mg **14** (0.47 mmol), catalyzed by 6 mg *t*Bu_3_P Pd G3 (9 µmol) in 6.0 mL dioxane and 1.9 mL of a 0.5 M K_2_CO_3_ solution. The reaction was conducted at ambient temperature and the crude product was purified via flash chromatography (DCM/MeOH (0–5%)). Yield: 90 mg (63%) as a white solid. ^1^H NMR (200 MHz, CDCl_3_ + MeOD) δ 9.44 (s, 1H), 8.23 (s, 1H), 8.19 (s, 1H), 7.75 (s, 1H), 7.24 (d, *J* = 9.3 Hz, 2H), 7.04 (dd, *J* = 6.9, 3.8 Hz, 2H), 6.57 (dt, *J* = 28.2, 8.8 Hz, 1H), 6.14 (s, 1H), 6.11 (s, 1H), 5.45 (t, *J* = 5.8 Hz, 1H), 5.32 (s, 2H), 3.26 (t, *J* = 8.1 Hz, 2H), 1.90 (s, 3H), 0.60 (t, *J* = 8.2 Hz, 2H), −0.37 (s, 9H). ^13^C NMR (50 MHz, CDCl_3_ + MeOD) δ 170.1, 164.7, 145.4, 138.6, 137.0, 134.7, 131.2, 129.5, 129.3, 127.2, 126.0, 122.2, 120.5, 118.4, 118.3, 117.8, 115.7, 73.1, 66.3, 23.4, 17.6, −1.7. TLC-MS (ESI) *m*/*z*: 473.1 [M + Na]^+^ HPLC t_ret_ = 9.18 min.

*N-(3-(5-(2-cyclopentylacetamido)-1-((2-(trimethylsilyl)ethoxy)methyl)-1H-pyrrolo[2,3-b]pyridin-3-yl)phenyl)acrylamide* (**34g**): The preparation was performed following General Procedure A from 120 mg **33g** (0.27 mmol) and 109 mg **14** (0.40 mmol), catalyzed by 5 mg tBu_3_P Pd G3 (8 µmol) in 6.0 mL dioxane and 1.6 mL of a 0.5 M K_2_CO_3_ solution. The reaction was conducted at ambient temperature and the crude product was purified via flash chromatography (DCM/MeOH (0–5%)). Yield: 85 mg (62%) as a white solid. ^1^H NMR (200 MHz, DMSO-*d*_6_) δ 10.26 (s, 1H), 10.04 (s, 1H), 8.54 (s, 1H), 8.47 (s, 1H), 8.01 (s, 1H), 7.94 (s, 1H), 7.58 (d, *J* = 6.7 Hz, 1H), 7.53–7.34 (m, 2H), 6.68–6.37 (m, 1H), 6.27 (d, *J* = 17.8 Hz, 1H), 5.78 (d, *J* = 12.2 Hz, 1H), 5.65 (s, 2H), 4.12 (t, 2H), 2.34 (s, 2H), 1.67 (d, *J* = 38.3 Hz, 5H), 1.23 (s, 4H), 0.84 (t, 2H), −0.10 (s, 9H). ^13^C NMR (50 MHz, CDCl_3_ + MeOD) δ 172.8, 164.7, 145.3, 138.8, 136.7, 134.8, 131.3, 129.7, 129.5, 127.4, 126.0, 122.2, 120.5, 118.6, 118.3, 117.9, 115.8, 73.2, 66.4, 43.2, 37.2, 32.5, 24.9, 17.8, −1.6. TLC-MS (ESI) *m*/*z*: 541.5 [M + Na]^+^ HPLC t_ret_ = 10.60 min.

*N-(3-(5-(2-phenylacetamido)-1-((2-(trimethylsilyl)ethoxy)methyl)-1H-pyrrolo[2,3-b]pyridin-3-yl)phenyl)acrylamide* (**34h**): The preparation was performed following General Procedure A from 111 mg **33h** (0.24 mmol) and 98 mg **14** (0.36 mmol), catalyzed by 4 mg tBu_3_P Pd G3 (7 µmol) in 5.0 mL dioxane and 1.4 mL of a 0.5 M K_2_CO_3_ solution. The reaction was conducted at ambient temperature and the crude product was purified via flash chromatography (petrol ether/EtOAc + 10% MeOH (20–60%)). Yield: 59 mg (47%) as a white solid. ^1^H NMR (200 MHz, CDCl_3_ + MeOD) δ 9.79 (s, 1H), 9.72 (s, 1H), 8.53 (d, *J* = 2.3 Hz, 1H), 8.37 (s, 1H), 7.85 (s, 1H), 7.50 (s, 1H), 7.47 (s, 1H), 7.31 (d, *J* = 5.3 Hz, 1H), 7.28–7.18 (m, 2H), 7.24–7.07 (m, 2H), 7.10–6.81 (m, 1H), 6.32 (s, 1H), 6.29 (s, 1H), 6.24 (d, *J* = 5.8 Hz, 1H), 5.62 (dt, *J* = 11.8, 6.3 Hz, 1H), 5.54 (s, 2H), 4.22 (s, 2H), 3.44 (t, 2H), 0.78 (t, 2H), −0.19 (s, 9H). ^13^C NMR (50 MHz, CDCl_3_ + MeOD) δ 170.8, 164.8, 144.9, 138.6, 136.2, 134.8, 134.5, 131.1, 129.6, 129.3, 129.0, 128.5, 127.1, 126.9, 126.2, 122.3, 120.8, 118.7, 118.3, 118.0, 115.9, 73.2, 66.3, 43.6, 17.6, −1.9. TLC-MS (ESI) *m*/*z*: 549.1 [M + Na]^+^ HPLC t_ret_ = 10.02 min.

*N-(3-(5-(2-(thiophen-2-yl)acetamido)-1-((2-(trimethylsilyl)ethoxy)methyl)-1H-pyrrolo[2,3-b]pyridin-3-yl)phenyl)acrylamide* (**34i**): The preparation was performed following General Procedure A from 123 mg **33i** (0.26 mmol) and 108 mg **14** (0.39 mmol), catalyzed by 5 mg tBu_3_P Pd G3 (8 µmol) in 6.0 mL dioxane and 1.6 mL of a 0.5 M K_2_CO_3_ solution. The reaction was conducted at ambient temperature and the crude product was purified via flash chromatography (DCM/MeOH (0–5%)). Yield: 87 mg (62%) as a white solid. ^1^H NMR (200 MHz, CDCl_3_) δ 8.62 (d, *J* = 2.3 Hz, 1H), 8.49 (d, *J* = 2.3 Hz, 1H), 8.35 (s, 1H), 8.23 (s, 1H), 8.05 (s, 1H), 7.73 (s, 1H), 7.68 (s, 1H), 7.52 (d, *J* = 3.3 Hz, 4H), 7.28 (s, 1H), 6.69 (dd, *J* = 16.6, 1.8 Hz, 1H), 6.55 (dd, *J* = 16.7, 9.4 Hz, 1H), 5.97 (dd, *J* = 9.5, 1.8 Hz, 1H), 5.84 (s, 2H), 4.21 (s, 2H), 3.79 (t, *J* = 8.1 Hz, 2H), 1.16 (t, *J* = 8.2 Hz, 2H), 0.19 (s, 9H). ^13^C NMR (50 MHz, CDCl_3_) δ 168.8, 146.0, 142.7, 141.4, 138.3, 137.7, 135.6, 134.9, 129.4, 128.2, 127.6, 127.4, 126.2, 125.8, 121.2, 118.2, 118.0, 115.9, 92.4, 84.8, 84.3, 73.0, 66.3, 38.0, 17.7, −1.5. TLC-MS (ESI) *m*/*z*: 555.1 [M + Na]^+^ HPLC t_ret_ = 9.71 min.

*N-(3-(3-acrylamidophenyl)-1H-pyrrolo[2,3-b]pyridin-5-yl)cyclopentanecarboxamide* (**12a**): The preparation was carried out following *General Procedure B* starting from 70 mg **34a** (0.14 mmol) in 5 mL DCM and 2 mL TFA. Flash purification (DCM/MeOH (2–8%)) afforded 40 mg (77%) of the final compound as a white solid. ^1^H NMR (200 MHz, DMSO) δ 11.84 (br s, 1H), 10.24 (s, 1H), 9.97 (s, 1H), 8.62–8.47 (m, 1H), 8.47–8.32 (m, 1H), 7.93 (s, 1H), 7.75 (d, *J* = 2.2 Hz, 1H), 7.68–7.54 (m, 1H), 7.49–7.26 (m, 2H), 6.49 (dd, *J* = 16.9, 9.9 Hz, 1H), 6.28 (dd, *J* = 16.9, 1.6 Hz, 1H), 5.78 (dd, *J* = 9.9, 1.6 Hz, 1H), 2.91–2.71 (m, 1H), 1.99–1.46 (m, 8H). ^13^C NMR (50 MHz, DMSO) δ 174.5, 163.3, 145.7, 139.5, 136.8, 135.6, 132.0, 129.7, 129.3, 126.9, 124.4, 121.7, 118.3, 117.3, 117.0, 116.7, 114.3, 45.1, 30.2, 25.8. TLC-MS (ESI) *m*/*z*: 397.0 [M + Na]^+^ HPLC t_ret_ = 7.38 min.

*N-(3-(3-acrylamidophenyl)-1H-pyrrolo[2,3-b]pyridin-5-yl)thiophene-2-carboxamide* (**12c**): The preparation was carried out following *General Procedure B* starting from 46 mg **34c** (0.09 mmol) in 4.2 mL DCM and 1.8 mL TFA. Flash purification (DCM/MeOH (4–10%)) afforded 15 mg (44%) of the final compound as a white solid. ^1^H NMR (200 MHz, DMSO-*d*_6_) δ 11.94 (s, 1H), 10.42 (s, 1H), 10.27 (s, 1H), 8.55 (s, 2H), 8.06 (d, *J* = 3.7 Hz, 1H), 7.97 (s, 1H), 7.87 (d, *J* = 5.1 Hz, 1H), 7.82 (s, 1H), 7.65 (dd, *J* = 5.8, 2.1 Hz, 1H), 7.41 (s, 1H), 7.39 (s, 1H), 7.25 (t, *J* = 4.4 Hz, 1H), 6.49 (dd, *J* = 16.9, 9.8 Hz, 1H), 6.27 (dd, *J* = 17.0, 1.8 Hz, 1H), 5.77 (dd, *J* = 9.8, 2.2 Hz, 1H). ^13^C NMR (50 MHz, DMSO-*d*_6_) δ 163.2, 160.1, 146.2, 139.9, 139.5, 138.0, 135.4, 131.9, 131.7, 129.3, 129.0, 128.7, 128.1, 126.9, 124.6, 121.6, 120.2, 117.2, 116.9, 116.7, 114.3. TLC-MS (ESI) *m*/*z*: 411.3 [M + Na]^+^. HRMS ESI-TOF [M + H]^+^ *m*/*z* calcd. for C_21_H_16_N_4_O_2_S: 389.1067, found: 389.1072. HPLC t_ret_ = 6.44 min.

*N-(3-(3-acrylamidophenyl)-1H-pyrrolo[2,3-b]pyridin-5-yl)furan-2-carboxamide* (**12d**): The preparation was carried out following *General Procedure B* starting from 58 mg **34d** (0.12 mmol) in 4.2 mL DCM and 1.8 mL TFA. Flash purification (DCM/MeOH (4–10%)) afforded 23 mg (54%) of the final compound as a white solid. ^1^H NMR (200 MHz, DMSO-*d*_6_) δ 11.92 (s, 1H), 10.34 (s, 1H), 10.22 (s, 1H), 8.57 (s, 2H), 7.96 (s, 2H), 7.86–7.74 (m, 1H), 7.62 (d, *J* = 5.3 Hz, 1H), 7.50–7.33 (m, 2H), 7.33 (d, *J* = 3.5 Hz, 1H), 6.72 (dd, *J* = 3.2, 1.4 Hz, 1H), 6.48 (dd, *J* = 17.0, 9.8 Hz, 1H), 6.29 (dd, 1H), 5.77 (dd, *J* = 9.6, 2.0 Hz, 1H). ^13^C NMR (50 MHz, DMSO-*d*_6_) δ 163.2, 156.5, 147.6, 146.2, 145.6, 139.5, 138.0, 135.4, 131.9, 129.3, 128.4, 126.9, 124.5, 121.6, 120.2, 117.2, 116.9, 116.7, 114.6, 114.2, 112.1. TLC-MS (ESI) *m*/*z*: 395.2 [M + Na]^+^. HRMS ESI-TOF [M + H]^+^ *m*/*z* calcd. for C_21_H_16_N_4_O_3_: 373.1295, found: 373.1300. HPLC t_ret_ = 5.88 min

*N-(3-(3-acrylamidophenyl)-1H-pyrrolo[2,3-b]pyridin-5-yl)benzamide* (**12e**): The preparation was carried out following *General Procedure B* starting from 47 mg **34e** (0.09 mmol) in 4.2 mL DCM and 1.8 mL TFA. Flash purification (DCM/MeOH (4–10%)) afforded 10 mg (29%) of the final compound as a white solid. ^1^H NMR (400 MHz, DMSO-*d*_6_) δ 11.92 (d, 1H), 10.39 (s, 1H), 10.23 (s, 1H), 8.62 (d, *J* = 2.3 Hz, 1H), 8.59 (d, *J* = 2.2 Hz, 1H), 8.03 (s, 1H), 8.01 (d, *J* = 1.6 Hz, 1H), 7.96 (s, 1H), 7.81 (d, *J* = 2.6 Hz, 1H), 7.66–7.61 (m, 1H), 7.60 (d, *J* = 6.7 Hz, 1H), 7.55 (t, *J* = 7.2 Hz, 2H), 7.41 (d, *J* = 6.5 Hz, 2H), 6.47 (dd, *J* = 16.9, 10.1 Hz, 1H), 6.27 (dd, *J* = 17.0, 2.1 Hz, 1H), 5.76 (dd, *J* = 10.0, 2.1 Hz, 1H). ^13^C NMR (101 MHz, DMSO-*d*_6_) δ 165.5, 163.2, 146.1, 139.5, 138.0, 135.4, 134.6, 131.9, 131.5, 129.2, 129.1, 128.3, 127.5, 126.7, 124.4, 121.6, 120.0, 117.2, 116.9, 116.6, 114.3. TLC-MS (ESI) *m*/*z*: 405.3 [M + Na]^+^. HRMS ESI-TOF [M + H]^+^ *m*/*z* calcd. for C_23_H_18_N_4_O_2_: 383.1503, found: 383.1509. HPLC t_ret_ = 6.74 min.

*N-(3-(5-acetamido-1H-pyrrolo[2,3-b]pyridin-3-yl)phenyl)acrylamide* (**12f**): The preparation was carried out following *General Procedure B* starting from 90 mg **34f** (0.20 mmol) in 4.2 mL DCM and 1.8 mL TFA. Flash purification (DCM/MeOH (10–16%)) afforded 26 mg (41%) of the final compound as a white solid. ^1^H NMR (200 MHz, DMSO-*d*_6_) δ 11.85 (s, 1H), 10.22 (s, 1H), 10.03 (s, 1H), 8.46 (s, 1H), 8.39 (s, 1H), 7.95 (s, 1H), 7.76 (s, 1H), 7.60 (d, *J* = 7.3 Hz, 1H), 7.38 (q, *J* = 7.7 Hz, 2H), 6.49 (dd, *J* = 16.9, 9.8 Hz, 1H), 6.28 (d, *J* = 16.5 Hz, 1H), 5.78 (d, *J* = 9.7 Hz, 1H), 2.08 (s, 3H). ^13^C NMR (50 MHz, DMSO-*d*_6_) δ 168.3, 163.2, 145.8, 139.5, 136.8, 135.5, 131.9, 129.4, 129.3, 126.9, 124.4, 121.6, 118.4, 117.2, 116.9, 116.7, 114.2, 23.7. TLC-MS (ESI) *m*/*z*: 343.1 [M + Na]^+^. HRMS ESI-TOF [M + H]^+^ *m*/*z* calcd. for C_18_H_16_N_4_O_2_: 321.1346, found: 321.1352. HPLC t_ret_ = 4.74 min.

*N-(3-(5-(2-cyclopentylacetamido)-1H-pyrrolo[2,3-b]pyridin-3-yl)phenyl)acrylamide* (**12g**): The preparation was carried out following *General Procedure B* starting from 85 mg **34g** (0.16 mmol) in 7 mL DCM and 3 mL TFA. Flash purification (DCM/MeOH (5–6%)) afforded 27 mg (42%) of the final compound as a white solid. ^1^H NMR (400 MHz, DMSO-*d*_6_) δ 11.84 (s, 1H), 10.22 (s, 1H), 9.94 (s, 1H), 8.49 (d, *J* = 2.2 Hz, 1H), 8.42 (d, *J* = 2.2 Hz, 1H), 7.96 (s, 1H), 7.76 (d, *J* = 2.4 Hz, 1H), 7.60 (d, *J* = 8.2 Hz, 1H), 7.41 (t, *J* = 7.8 Hz, 1H), 7.35 (d, *J* = 7.7 Hz, 1H), 6.48 (dd, *J* = 16.9, 10.1 Hz, 1H), 6.29 (dd, *J* = 17.0, 2.0 Hz, 1H), 5.78 (dd, *J* = 10.0, 2.0 Hz, 1H), 2.35 (s, 1H), 2.33 (s, 1H), 2.27 (dt, *J* = 15.1, 7.4 Hz, 1H), 1.78 (dq, *J* = 11.8, 6.2 Hz, 2H), 1.62 (pd, *J* = 9.0, 8.3, 5.0 Hz, 2H), 1.59–1.45 (m, 2H), 1.22 (dq, *J* = 11.5, 7.3 Hz, 2H). ^13^C NMR (101 MHz, DMSO-*d*_6_) δ 171.0, 163.3, 145.9, 139.6, 137.0, 135.7, 132.1, 129.6, 129.3, 126.9, 124.4, 121.8, 118.6, 117.5, 117.1, 116.8, 114.4, 42.5, 36.8, 32.1, 24.7. TLC-MS (ESI) *m*/*z*: 411.4 [M + Na]^+^. HRMS ESI-TOF [M + H]^+^ *m*/*z* calcd. for C_23_H_24_N_4_O_2_: 389.1972, found: 389.1977. HPLC t_ret_ = 7.85 min.

*N-(3-(5-(2-phenylacetamido)-1H-pyrrolo[2,3-b]pyridin-3-yl)phenyl)acrylamide* (**12h**): The preparation was carried out following *General Procedure B* starting from 59 mg **34h** (0.11 mmol) in 4.2 mL DCM and 1.8 mL TFA. Flash purification (DCM/MeOH (4–10%)) afforded 15 mg (34%) of the final compound as a white solid. ^1^H NMR (400 MHz, DMSO-*d*_6_) δ 11.86 (s, 1H), 10.28 (s, 1H), 10.22 (s, 1H), 8.51 (d, *J* = 2.3 Hz, 1H), 8.43 (d, *J* = 2.3 Hz, 1H), 7.93 (t, *J* = 1.9 Hz, 1H), 7.76 (d, *J* = 2.3 Hz, 1H), 7.60 (dt, *J* = 8.1, 1.6 Hz, 1H), 7.36 (ddt, *J* = 14.8, 12.3, 7.7 Hz, 6H), 7.28–7.22 (m, 1H), 6.47 (dd, *J* = 17.0, 10.1 Hz, 1H), 6.28 (dd, *J* = 17.0, 2.1 Hz, 1H), 5.77 (dd, *J* = 10.1, 2.1 Hz, 1H), 3.68 (s, 2H). ^13^C NMR (101 MHz, DMSO-*d*_6_) δ 169.0, 163.1, 145.8, 139.4, 136.6, 135.9, 135.4, 131.9, 129.4, 129.1, 129.1, 128.2, 126.7, 126.4, 124.3, 121.6, 118.3, 117.2, 116.9, 116.6, 114.2, 43.0. TLC-MS (ESI) *m*/*z*: 419.4 [M + Na]^+^. HRMS ESI-TOF [M + H]^+^ *m*/*z* calcd. for C_24_H_20_N_4_O_2_: 397.1659, found: 397.1662. HPLC t_ret_ = 6.98 min.

*N-(3-(5-(2-(thiophen-2-yl)acetamido)-1H-pyrrolo[2,3-b]pyridin-3-yl)phenyl)acrylamide* (**12i**): The preparation was carried out following *General Procedure B* starting from 87 mg **34i** (0.16 mmol) in 4.2 mL DCM and 1.8 mL TFA. Flash purification (DCM/MeOH (6–8%)) afforded 15 mg (34%) of the final compound as a white solid. ^1^H NMR (200 MHz, DMSO-*d*_6_) δ 11.88 (s, 1H), 10.33 (s, 1H), 10.23 (s, 1H), 8.49 (s, 1H), 8.43 (s, 1H), 7.94 (s, 1H), 7.77 (s, 1H), 7.61 (d, *J* = 6.8 Hz, 1H), 7.38 (d, *J* = 7.9 Hz, 3H), 7.01 (s, 2H), 6.48 (dd, 1H), 6.27 (d, *J* = 17.2 Hz, 1H), 5.78 (d, *J* = 9.6 Hz, 1H), 3.92 (s, 2H). ^13^C NMR (50 MHz, DMSO-*d*_6_) δ 168.1, 163.2, 145.9, 139.5, 137.1, 136.6, 135.4, 131.9, 129.2, 129.2, 126.9, 126.6, 126.3, 125.0, 124.5, 121.6, 118.4, 117.2, 116.9, 116.7, 114.2, 37.2. TLC-MS (ESI) *m*/*z*: 425.2 [M + Na]^+^. HRMS ESI-TOF [M + H]^+^ *m*/*z* calcd. for C_22_H_18_N_4_O_2_S: 403.1223, found: 403.1227. HPLC t_ret_ = 6.71 min.

*N-(3-(1H-pyrrolo[2,3-b]pyridin-3-yl)phenyl)propionamide* (**9b**): To a solution of 17 mg **9a** (0.07 mmol) in THF/MeOH (6 mL each) were added 8 mg Pd/C and the reaction was purged with hydrogen. The mixture was then stirred under hydrogen until HPLC indicated complete conversion. The catalyst was filtered off, the filtrate evaporated and the residue purified via flash chromatography (DCM/MeOH (4–8%)). Yield: 12 mg (69%) as a white solid. ^1^H NMR (200 MHz, DMSO) δ 11.89 (br s, 1H), 9.90 (s, 1H), 8.37–8.19 (m, 2H), 8.10–7.98 (m, 1H), 7.87–7.75 (m, 1H), 7.56–7.41 (m, 1H), 7.41–7.28 (m, 2H), 7.25–7.06 (m, 1H), 2.35 (q, *J* = 7.4 Hz, 2H), 1.11 (t, *J* = 7.4 Hz, 3H) ^13^C NMR (50 MHz, DMSO) δ 172.1, 149.1, 142.9, 139.8, 135.3, 129.1, 127.4, 123.6, 120.8, 117.2, 116.8, 116.3, 116.0, 114.2, 29.6, 9.7 TLC-MS (ESI) *m*/*z*: 266.4 [M + H]^+^ HPLC t_ret_ = 5.47 min.

*3-(3-propionamidophenyl)-1H-pyrrolo[2,3-b]pyridine-5-carboxamide* (**10b**): To a solution of 25 mg **10a** (0.08 mmol) in THF/MeOH (5 mL each) were added 8 mg Pd/C and the reaction was purged with hydrogen. The mixture was then stirred under hydrogen until HPLC indicated complete conversion. The catalyst was filtered off, the filtrate evaporated and the residue purified via flash chromatography (DCM/MeOH (6–16%)). Yield: 15 mg (60%) as a white solid. ^1^H NMR (400 MHz, DMSO) δ 12.15 (s, 1H), 9.95 (s, 1H), 8.81 (s, *J* = 24.3 Hz, 1H), 8.75 (s, 1H), 8.10 (br s, 1H), 7.88 (d, *J* = 19.3 Hz, 2H), 7.61 (d, *J* = 7.2 Hz, 1H), 7.46–7.25 (m, 3H), 2.36 (q, *J* = 7.5 Hz, 2H), 1.11 (t, *J* = 7.5 Hz, 3H) ^13^C NMR (100 MHz, DMSO) δ 172.0, 167.7, 150.1, 143.2, 139.8, 134.8, 129.1, 127.2, 124.7, 122.6, 121.3, 117.1, 116.9, 116.4, 115.4, 29.5, 9.6 TLC-MS (ESI) *m*/*z*: 331.4 [M + Na]^+^ HPLC t_ret_ = 3.94 min.

*N-cyclopentyl-3-(3-propionamidophenyl)-1H-pyrrolo[2,3-b]pyridine-5-carboxamide* (**11b**): To a solution of 20 mg **11a** (0.05 mmol) in THF/MeOH (2 mL each) were added 8 mg Pd/C and the reaction was purged with hydrogen. The mixture was then stirred under hydrogen until HPLC indicated complete conversion. The catalyst was filtered off, the filtrate evaporated and the residue purified via flash chromatography (DCM/MeOH (6–14%)). Yield: 19 mg (95%) as a white solid. ^1^H NMR (400 MHz, DMSO) δ 12.15 (br s, 1H), 9.98 (s, 1H), 8.76 (d, *J* = 1.6 Hz, 1H), 8.69 (d, *J* = 1.6 Hz, 1H), 8.39 (d, *J* = 7.2 Hz, 1H), 7.94 (s, 1H), 7.86 (d, *J* = 2.3 Hz, 1H), 7.62–7.55 (m, 1H), 7.43–7.32 (m, 2H), 4.33–4.21 (m, 1H), 2.36 (q, *J* = 7.5 Hz, 2H), 1.96–1.86 (m, 2H), 1.76–1.65 (m, 2H), 1.62–1.49 (m, 4H), 1.11 (t, *J* = 7.5 Hz, 3H). ^13^C NMR (101 MHz, DMSO) δ 172.0, 165.7, 149.9, 142.8, 139.8, 134.8, 129.1, 126.9, 124.7, 123.2, 121.2, 117.2, 116.8, 116.3, 115.4, 50.9, 32.1, 29.5, 23.6, 9.6. TLC-MS (ESI) *m*/*z*: 399.1 [M + Na]^+^ HPLC t_ret_ = 7.15 min.

*N-(3-(3-propionamidophenyl)-1H-pyrrolo[2,3-b]pyridin-5-yl)cyclopentanecarboxamide* (**12b**): To a solution of 18 mg **12a** (0.05 mmol) in THF/MeOH (2 mL each) were added 5 mg Pd/C and the reaction was purged with hydrogen. The mixture was then stirred under hydrogen until HPLC indicated complete conversion. The catalyst was filtered off, the filtrate evaporated and the residue purified via flash chromatography (DCM/MeOH (3–10%)). Yield: 15 mg (83%) as a white solid. ^1^H NMR (200 MHz, DMSO) δ 11.82 (br s, 1H), 10.05–9.81 (m, 2H), 8.50 (d, *J* = 1.7 Hz, 1H), 8.41 (d, *J* = 1.7 Hz, 1H), 7.90–7.78 (m, 1H), 7.72 (d, *J* = 1.8 Hz, 1H), 7.63–7.46 (m, 1H), 7.44–7.20 (m, *J* = 7.7 Hz, 2H), 2.91–2.72 (m, 1H), 2.35 (q, *J* = 7.4 Hz, 2H), 1.97–1.50 (m, 8H), 1.10 (t, *J* = 7.4 Hz, 3H). TLC-MS (ESI) *m*/*z*: 399.2 [M + Na]^+^ HPLC t_ret_ = 7.40 min.

### 4.2. JAK3 Crystal Structure Determination

Recombinant JAK3 kinase domain was expressed and purified as described previously [21,22]. The protein (10 mg/mL) was incubated with the inhibitors at 1:1.2 molar ratio, and the complexes were crystallized using sitting drop vapor diffusion method at 4 °C and the conditions containing 25% PEG 3350, 0.1–0.2 M MgCl_2_ and 0.1 M MES, pH 5.5. The crystals were cryo-protected using mother liquor supplemented with 20% ethylene glycol, and diffraction data were collected at SLS X06SA. The data were processed and scaled with XDS [40] and Aimless [41], respectively. Molecular replacement using Phaser [42] and the coordinate of JAK3 (pdb id: 5lwm) was performed. Model rebuilding alternated with refinement was performed in COOT [43] and REFMAC [44], respectively. Data collection and refinement statistics are summarized in Table 7.

### 4.3. Biological Assays

#### 4.3.1. Thermal Shift Assay

The kinase domains of JAK3 and BMX at 2 μM were mixed with the inhibitors at 10 μM, and subsequently SyPRO orange dye (Invitrogen Carlsbad, CA, USA) was added. The thermal shift assay and data evaluation were performed as described previously using a Real-Time PCR Mx3005p machine (Stratagene, La Jolla, CA, USA) [45,46].

#### 4.3.2. Enzymatic Activity Assay

If not mentioned differently, in vitro profiling of compounds was performed at Reaction Biology Corporation using the HotSpot™ assay platform. IC_50_ values were determined as singlicates using five doses with 5- or 10-fold serial dilution starting at 0.5 µM, 1 µM or 10 μM. Further details on the assay can be found on the supplier homepage (http://www.reactionbiology.com; see also Anastassiadis et al. 2011 [31]).

#### 4.3.3. Cellular NanoBRET Assay

Full length BMX and BTK was cloned into pFC32K (Promega, Madison, WI, USA) for expression of a C-terminal NanoLuc fusion. The plasmids were transfected into HEK293T cells cultured in DMEM (Gibco, Waltham, MA, USA) supplemented with 10% fetal bovine serum (Gibco, Waltham, MA, USA) and Penicillin/Streptamycin (Gibco, Waltham, MA, USA). NanoBRET assays were performed using the protocol published previously [47]. Briefly, after transfection and 20 h incubation cells were harvested and subsequently resuspended in OptiMEM (Gibco, Waltham, MA, USA). Cells were aliquoted onto 1534-well plates (Greiner, Kremsmünster, Austria), and inhibitors as well as 0.5 µM Tracer K4 (Promega) for BMX or 0.5 µM Tracer K5 (Promega, Madison, WI, USA) for BTK were added using an ECHO acoustic dispenser. The plates were incubated at 37 °C with 5% CO_2_ for 2 h prior to the addition of both NanoBRET NanoGlo substrate (Promega, Madison, WI, USA) and extracellular NanoLuc inhibitor. BRET luminescence (450 nm for donor emission and 610 nm for BRET signal) was measured using PHERAstar FSX plate reader (BMG Labtech, Ortenberg, Germany). Milli-BRET units (mBU) were calculated as a ratio between BRET signal and the overall measured luminescence. A dose–response fitting was applied and IC_50_ values were calculated using the Prism software. The affinity of both tracer molecules towards BMX and BTK was tested and showed similar values (EC_50_ (Tracer 4, **BMX**) = 0.240 µM, EC50 (Tracer 5, **BTK**) = 0.231 µM) indicating no bias of the assay due to the higher affinity of one kinase. Experiments were performed as tripicates and repeated at least three times.

### 4.4. Mass Spectrometric Investigation of Covalent Binding to BMX

The kinase domain of BMX was diluted to 0.05 mM with buffer containing 20 mM Tris pH 8.0, 200 mM NaCl, 0.5 mM TCEP (pH 7.0) and was subsequently mixed with 0.075 mM inhibitors (ratio of protein to inhibitor of 1:1.5). The mixture was incubated at 4°C. The samples were taken at different time points, the reaction stopped by diluting the sample with a 200-fold excess of 1% formic acid. The denatured protein and the adducts were assessed using electrospray time-of-flight (ESI-TOF) mass spectrometry.

### 4.5. Determination of Glutathione Reactivity

A total of 5 µL of a 10 mM compound stock solution in DMSO were added to 5 µL of a 4 mM Indoprofen solution in PBS as internal standard. The mixture was diluted with PBS buffer to a total volume of 1 mL of component A. Additionally, a freshly prepared solution of 10 mM GSH in PBS buffer was used as component B. A total of 250 µL of each component was mixed and immediately subjected to HPLC analysis for t^0^ measurement. The probes were stored at 40 °C in between the respective injections. t_1/2_ was determined by plotting natural logarithm of AUC/AUC_0_ against time.

### 4.6. Molecular Modeling

All the modelling was performed using the Schrödinger Small-Molecule Drug Discovery Suite 2019-1 (Schrödinger, LLC, New York, NY, USA). Noncovalent docking was performed with Glide in the XP mode after protein preparation using standard settings. Covalent docking was performed with the CovDock module in the pose prediction mode using standard settings. The figures were prepared with PyMOL 1.8.2.0. (Schrödinger, LLC, New York, NY, USA).

## Figures and Tables

**Table 1:** Selectivity of **9a** and **10a** in the JAK family and against EGFR.

Kinase	IC_50_ [nM] ^1^	
	9a	10a
JAK1	2970	460
JAK2	3580	1120
JAK3	0.2	<0.1
TYK2	5540	1900
EGFR	5250	>10,000

^1^ IC_50_ values were determined in a radiometric assay (Reaction Biology HotSpot™ [31]). Data were obtained as five-dose singlicate with 10-fold serial dilution starting at 10 μM. [ATP] = 10 μM.

**Table 2:** Selectivity of **9a** and **10a** in among the protein kinases with an equivalent cysteine.

Kinase	Gatekeeper Residue	Residual Activity @ 200 nM ^1^	
		9a	10a
BMX	T	2.7%	1.6%
TXK	T	1.8%	3.2%
BTK	T	18.2%	18.9%
TEC	T	37.1%	42.1%
ITK	**F**	88.1%	90.8%
JAK3	**M**	0.8%	0.5%
MKK7	**M**	37.3%	28.3%
EGFR	T	>100%	95.5%
HER2	T	95.0%	97.0%
HER4	T	66.1%	66.6%
BLK	T	95.8%	82.4%

^1^ Residual activities were determined in a radiometric assay (Reaction Biology HotSpot™ [31]). Data were obtained from duplicate measurements and reported as the arithmetic mean of the individual values. [ATP] = 10 μM.

**Table 3:** Inhibitory activity of acrylamides **10a** and **11a** and unreactive analog **11b**.

Kinase	IC_50_ [nM] ^1^		
	10a	11a	11b
BMX	29	539	>10,000
TXK	28	>1000 ^2^	>10,000
JAK3	<0.1	>10,000 ^3^	n.d.

^1^ IC_50_ values were determined in a radiometric assay (Reaction Biology HotSpot™ [31]). Data were obtained as five-dose singlicate with 10-fold serial dilution starting at 1 µM (for **10a**/**11a**) or 10 μM (**11b**). [ATP] = 10 μM. ^2^ 72% residual activity at 1 µM. ^3^ Determined in a JAK3 ELISA assay [33].

**Table 4:** Thermal stabilization of JAK3 and BMX by compound **10a** and *N*-alkylated analogs **11a** and **11c**–**g**.

	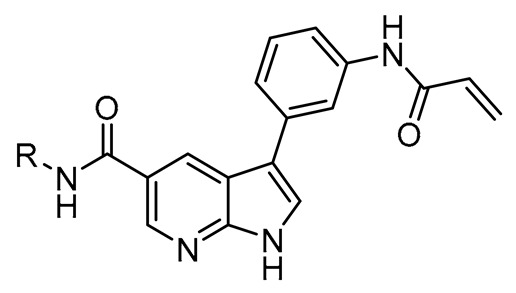		
Compound	R	JAK3 ΔT_m_ [K] ^1^	BMX ΔT_m_ [K] ^1^
**10a**	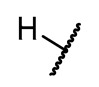	21.07 ± 0.03	10.49 ± 0.09
**11a**	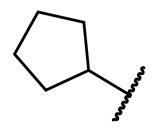	9.12 ± 0.02	5.31 ± 0.18
**11c**	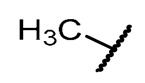	10.27 ± 0.37	7.84 ± 0.04
**11d**	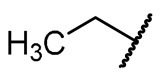	10.35 ± 0.26	7.35 ± 0.19
**11e**	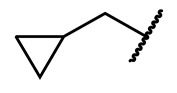	9.64 ± 0.28	6.94 ± 0.07
**11f**	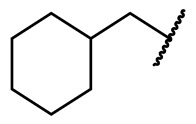	5.54 ± 0.82	3.68 ± 0.13
**11g**	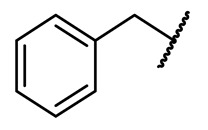	8.29 ± 1.26	6.84 ± 0.23

^1^ Mean of at least three independent measurements ± standard deviation.

**Table 5:** Thermal stabilization of compounds **12a** and **12c**–**i** and inhibitory potency of selected inhibitors on JAK3 and BMX.

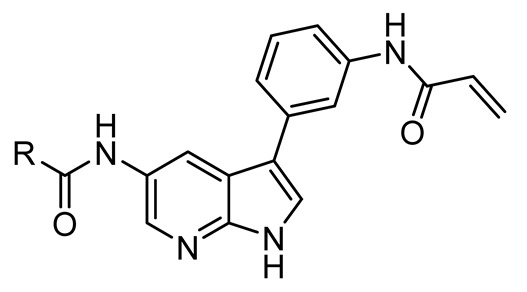					
Compound	R	JAK3 ΔT_m_ [K] ^1^	BMX ΔT_m_ [K] ^1^	IC_50_ JAK3 [nM] ^2^	IC_50_ BMX [nM] ^2^
12a	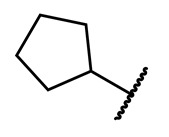	13.80 ± 0.13	11.92 ± 0.03	1340	2
12c	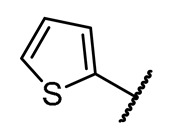	7.36 ± 0.44	11.52 ± 0.08	>10,000	1
12d	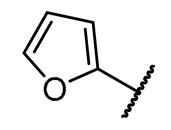	9.93 ± 0.12	10.75 ± 0.09	1080	4
12e	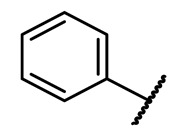	10.88 ± 0.95	11.37 ± 0.03	n.d.	n.d.
12f	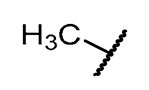	13.44 ± 0.43	10.57 ± 0.12	n.d.	n.d.
12g	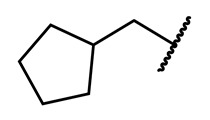	11.89 ± 0.23	11.88 ± 0.07	>10,000	6
12h	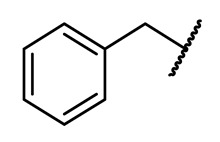	12.1	11.9	n.d.	n.d.
12i	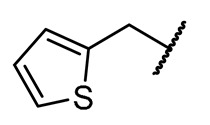	10.1	11.5	n.d.	16

^1^ Mean of at least three independent measurements ± standard deviation. ^2^ IC_50_ values were determined in a radiometric assay (Reaction Biology HotSpot™ [31]). Data were obtained as five-dose singlicate with 10-fold serial dilution starting at 10 µM (JAK3) or 1 μM (**12d**,**g**,**i** vs. BMX). For **12a**,**c** vs. BMX, 5-fold serial dilution starting from 0.5 µM was used. [ATP] to 10 μM.

**Table 6:** Inhibitory activity of compounds **12a–c** on all protein kinases with an αD-1 cysteine.

Kinase	Gatekeeper Residue	Residual Activity @ 200 nM ^1^			IC_50_ [nM] ^2^		
		12a	12b	12c	12a	12b	12c
BMX	T	4.8%	n.d.	0.2%	2.2	582	1.1
TXK	T	5.9%	88.8%	0.3%	13	n.d.	10
BTK	T	2.2%	69.7%	0.8%	2.2	n.d.	<1
TEC	T	25.4%	99.2%	17.2%	31	n.d.	13
ITK	**F**	98.5%	n.d.	94.0%	n.d.	n.d.	n.d
JAK3	**M**	94.5%	n.d.	>100%	1340	n.d.	>10,000
MKK7	**M**	100%	n.d.	>100%	n.d.	n.d.	n.d.
EGFR	T	51.3%	>100%	14.9%	158	n.d.	12
HER2	T	81.9%	>100%	41.1%	612	n.d.	118
HER4	T	15.9%	95.4%	4.7%	106	n.d.	8.5
BLK	T	14.7%	95.1%	8.6%	52	n.d.	30

^1^ Residual activities were determined in a radiometric assay (Reaction Biology HotSpot™ [31]). Data were obtained from duplicate measurements and reported as the arithmetic mean of the individual values. [ATP] = 10 μM. ^2^ IC_50_ values were determined in the above radiometric assay. Data were obtained as five-dose singlicate with 5-fold serial dilution starting at 0.5 μM (**12a**,**c** vs. BMX, BTK, TEC, TXK and HER4), 1 µM (**12a**,**c** vs. EGFR and HER2) or 10 µM (**12b** vs. BMX). A 10-fold serial dilution starting at 10 µM was employed for **12a**,**b** vs. JAK3. [ATP] = 10 μM.

**Table 7:** Data collection and refinement statistics for the JAK3-inhibitor complexes.

Complex	JAK3-9a	JAK3-10a
PDB Accession Code	7APG	7APF
*Data Collection*		
Resolution ^a^ (Å)	49.93–2.40 (2.53–2.40)	47.95–1.95 (2.02–1.95)
Spacegroup	*P*2_1_	*P*2_1_
Cell dimensions	*a* = 57.5, b = 112.9, *c* = 102.9 Å	*a* = 63.7, b = 62.5, *c* = 67.9 Å
	*α*, *γ* = 90.0°, *β* = 97.2	*α*, *γ* = 90.0°, *β* = 101.3°
No. unique reflections ^a^	50,511 (7245)	37,245 (3630)
Completeness ^a^ (%)	99.2 (98.1)	97.6 (97.7)
I/Σi ^a^	6.6 (1.8)	8.2 (2.4)
R_merge_^a^	0.126 (0.629)	0.108 (0.721)
CC (1/2) ^a^	0.994 (0.791)	0.994 (0.823)
Redundancy ^a^	4.6 (4.3)	6.0 (6.1)
*Refinement*		
No. atoms in refinement (P/L/O) ^b^	8952/100/449	4628/92/352
B factor (P/L/O) ^b^ (Å^2^)	40/16/37	33/20/36
R_fact_ (%)	21.2	20.4
R_free_ (%)	25.6	25.5
rms deviation bond ^c^ (Å)	0.010	0.013
rms deviation angle ^c^ (°)	1.1	1.3

^a^ Values in brackets show the statistics for the highest resolution shells. ^b^ P/L/O indicate protein, ligand molecules presented in the active sites, and other (water and solvent molecules), respectively. ^c^ rms indicates root-mean-square.

## Supplementary Materials

Supplementary Materials can be found at https://www.mdpi.com/1422-0067/21/23/9269/s1.

## Abbreviations

ATP	adenosine triphosphate
AUC	area under the curve
B_2_pin_2_	Bis(pinacolato)diboron
BLK	B-lymphoid tyrosine kinase
BMX/ETK	bone marrow tyrosine kinase on chromosome X
br s	broad singlet
BRET	bioluminescence resonance energy transfer
BTK	Bruton’s tyrosine kinase
CDI	1,1′carbonyldiimidazole
DAD	diode array detector
DCM	dichloromethane
DMEM	Dulbecco’s modified Eagle’s medium
DMF	*N*, *N*-dimethylformamide
DMSO	dimethyl sulfoxide
EC_50_	half maximal effective concentration
EGFR	epidermal growth factor receptor
ESI	electron spray ionization
Et_3_N	triethylamine
EtOAc	ethyl acetate
EtOH	ethanol
GSH	glutathione
HER	human epidermal growth factor receptor
HPLC	high performance liquid chromatography
IC_50_	half maximal inhibitory concentration
ITK	interleukin-2-inducible T-cell kinase
JAK	Janus kinase
KOAc	potassium acetate
LC	liquid chromatography
MAP2K7/MKK7	mitogen activated protein kinase kinase 7
MeOH	methanol
MES	2-(*N*-morpholino)ethanesulfonic acid
MS	mass spectrometry
NaH	sodium hydride
*n*-BuLi	*n*-Butyllithium
NBS	*N*-bromosuccinimide
NMR	nuclear magnetic resonance spectroscopy
Pd/C	palladium on activated carbon
Pd(OAc)_2_	palladium(II)acetate
PEG	polyethylene glycol
PH	pleckstrin homology
rt	room temperature
SAR	structure**–**activity relationship
sat.	saturated
SEM	2-(trimethylsilyl)ethoxymethyl
*t*BuOH	*tert*-Butanol
*t*Bu_3_P Pd G3	Mesyl[(tri-t-butylphosphine)-2-(2-aminobiphenyl)]palladium(II)
TCEP	tris(2-carboxyethyl)phosphine
∆T_m_	thermal shift
t_ret_	retention time
TEC	tyrosine kinase expressed in hepatocellular carcinoma
TFA	trifluoroacetic acid
TH	TEC homology
THF	tetrahydrofurane
TLC	thin layer chromatography
TMS	tetramethylsilane
TOF	time of flight
Tris	tris(hydroxymethyl)aminomethan
TsCl	tosyl chloride
TSK/EMT	interleukin-2-inducible T-cell kinase
TXK/RLK	tyrosine-protein kinase
UV	ultraviolet
XLA	X-linked agammaglobulinemia
XPhos	2-Dicyclohexylphosphino-2′,4′,6′-triisopropyl-1,1′-biphenyl
XPhos Pd G3	(2-Dicyclohexylphosphino-2′,4′,6′-triisopropyl-1,1′-biphenyl)[2-(2′-amino-1,1′-biphenyl)]palladium(II) methanesulfonate
XPhos Pd G4	(2-Dicyclohexylphosphino-2′,4′,6′-triisopropyl-1,1′-biphenyl)[2-(2′-*N*-methylamino-1,1′-biphenyl)]palladium(II) methanesulfonate
