# Supplementary material for: Discovery of a Novel Class of Covalent Dual Inhibitors Targeting the Protein Kinases BMX and BTK
Source: Int J Mol Sci. 2020 Dec 4;21(23):9269. doi: 10.3390/ijms21239269 (PMC7730235; doi:10.3390/ijms21239269)
Supplement: Supplementary file 1 [file ijms-21-09269-s001.pdf]

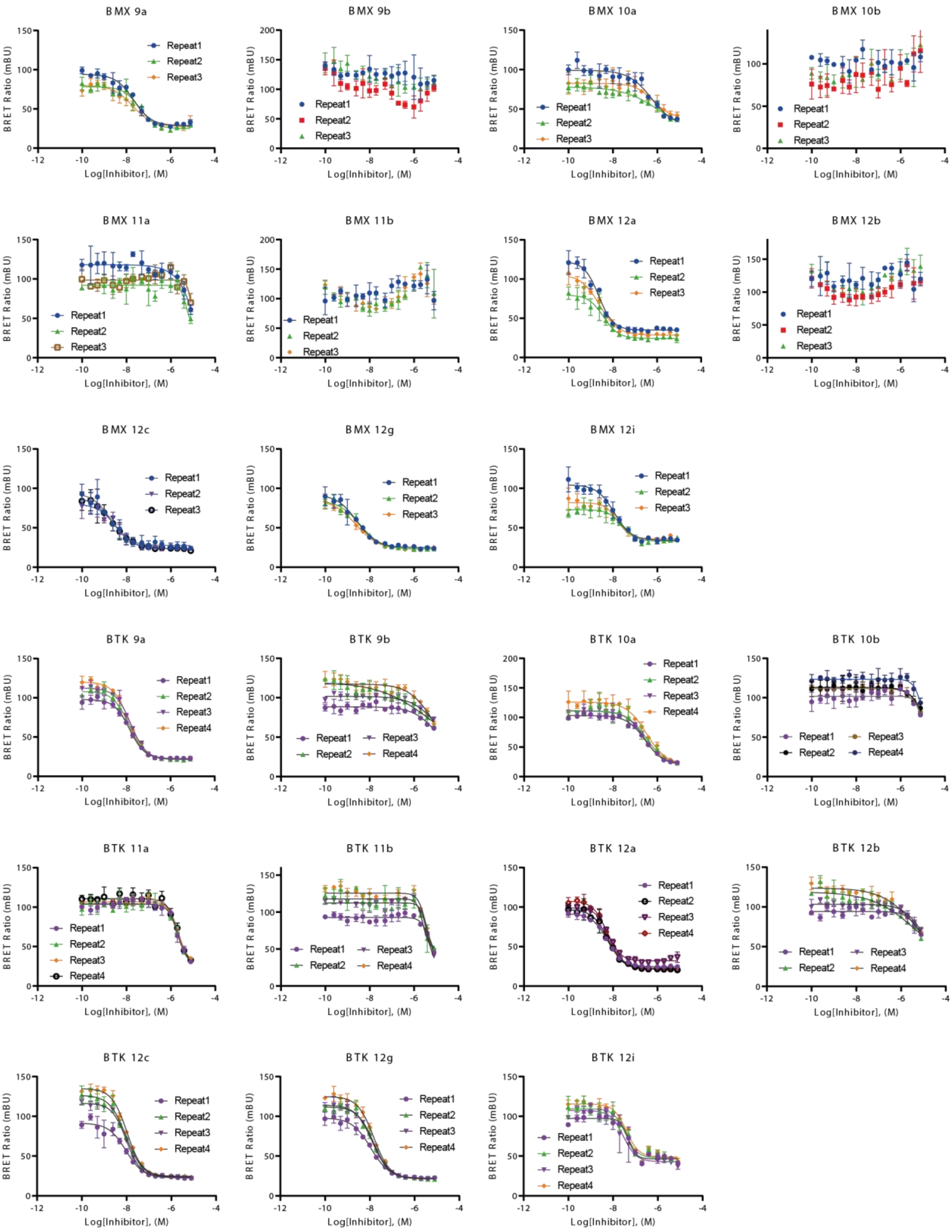

Supplementary figure 1: NanoBRET dose response curves of compounds titrated versus BMX and BTK. Each point is the average of three technical replicates and the standard deviation is shown. The curves of all biological repeats are depicted (BMX: n=3: BTK: n=4).

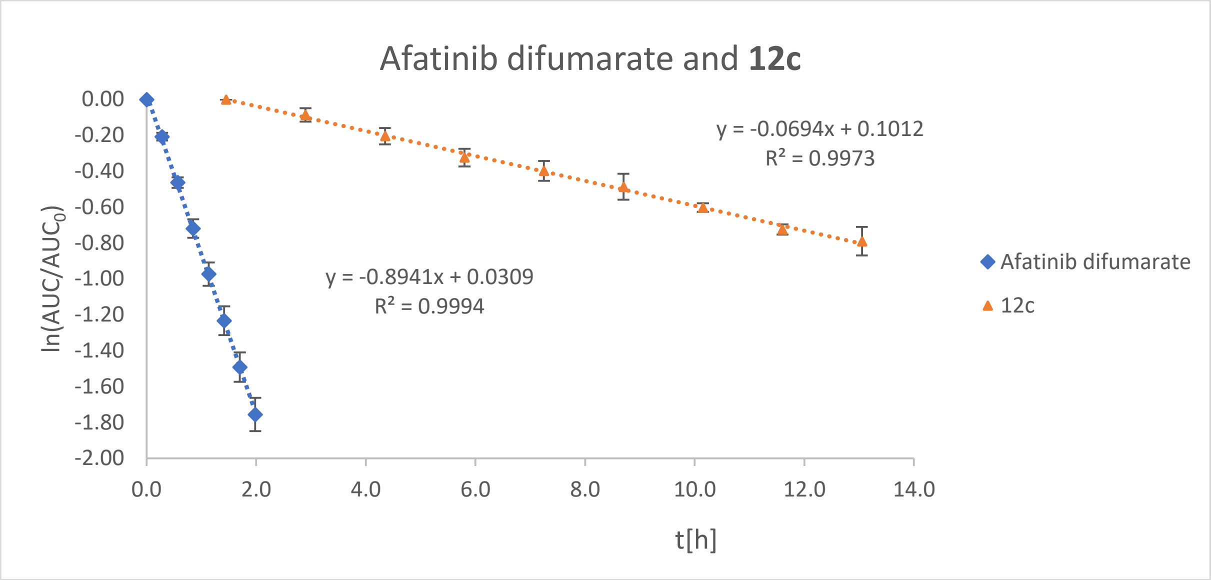

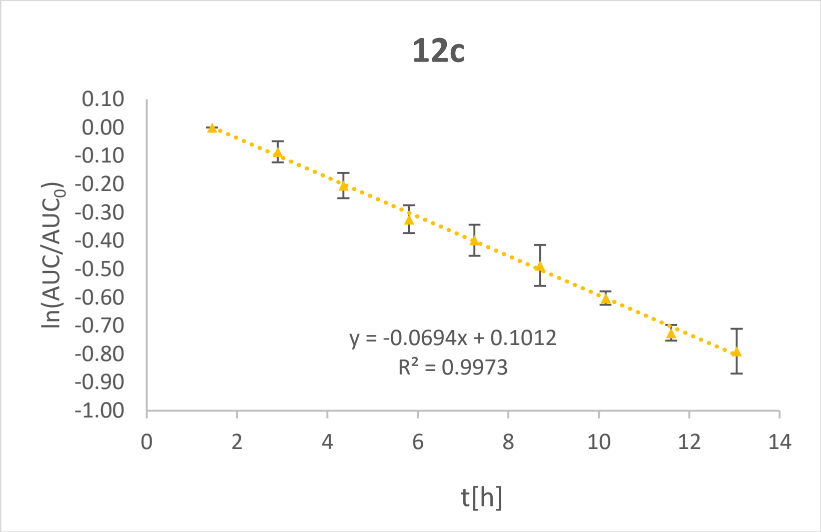

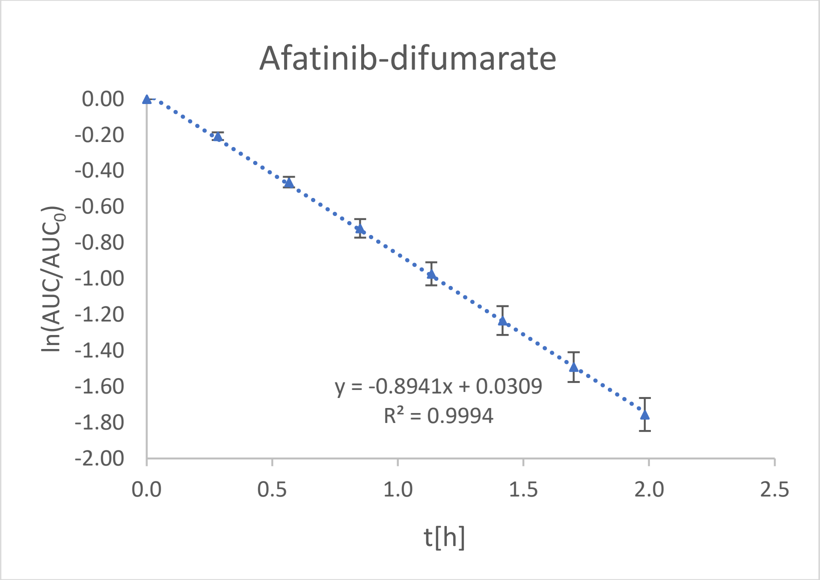

$t_{1/2}$ **12c:** 9,98 h
$t_{1/2}$ Afatinib: 0,76 h

Supplementary figure 2: Evaluation of stability against GSH (5 mM) at pH 7.4 for compound **12c** and Afatinib. Each point represents the average of three replicates and standard deviations are shown.

Compound	Compound Mol. Weight (Da)	30 min	60 min	120 min	240 min
**9a**	263.3	33557.6 Da (100%) +263.3 Da	33557.5 Da (100%) +263.3 Da	33557.2 Da (100%) +263.3 Da	33557.2 Da (100%) +263.3 Da
**9b**	265.32	33294.4 Da (100%) native	33294.2 Da (100%) native	33294.3 Da (100%) native	33293.8 Da (100%) native
**10a**	306.33	33600.5 Da (99%) +306.2 Da	33600.5 Da (100%) +306.3 Da	33600.2 (100%) +306.3 Da	33600.1 Da (100%) 306.2 Da
**10b**	308.34	33294.2 Da (100%) native	33294.3 (100%) native	33294.3 (100%) native	33293.9 (100%) native
**11a**	374.44	33294.3 Da (40%) native 33668.7 Da (60%) +374.4 Da	33294.6 Da (33%) native 33669.0 Da (67%) +374.8 Da	33295.0 Da (11%) native 33668.7 Da (89%) +373.8 Da	33295.1 Da (3%) native 33668.3 Da (97%) +374.4 Da
**11b**	376.46	33294.4 Da (100%) native	33294.2 Da (100%) native	33293.9 Da (100%) native	33293.9 Da (100%) native
**12a**	374.44	33294.6 Da(3%) native 33669.0 Da (97%) +374.7 Da	33295.8 Da (3%) native 33668.9 (97%) +374.7 Da	33295.2 Da (1%) native 33668.3 (99%) +374.4 Da	33294.9 (1%) native 33668.3 (99%) +374.4 Da
**12b**	376.46	33294.4 Da (100%) native	33294.2 Da (100%) native	33294.0 Da (100%) native	33293.9 Da (100%) native
**12c**	388.45	33682.5 Da (100%) +388.2 Da	33682.4 Da (100%) +388.2 Da	33681.9 Da (100%) +388 Da	33682.1 Da (100%) +388.2 Da
**12g**	388.47	33682.5 Da (100%) +388.2	33682.5 Da (100%) +388.3 Da	33296.6 Da (1%) native 33682.0 Da (99%) +388.1 Da	33682.2 Da (100%) +388.3 Da
**12i**	402.47	33696.7 Da (100%) +402.4 Da	33696.7 Da (100%) +402.5 Da	33696.2 Da (100%) + 402.3 Da	33696.3 Da (100%) +402.4 Da
BMX native		33294.3 Da (100%)	33294.2 Da (100%)	33293.9 Da (100%)	33293.9 Da (100%)

Supplementary table 1: Covalent adduct formation between selected compounds and BMX evaluated by mass spectrometry.
